# Placental Mitochondrial Abnormalities in Preeclampsia

**DOI:** 10.1007/s43032-021-00464-y

**Published:** 2021-02-01

**Authors:** Philippe Vangrieken, Salwan Al-Nasiry, Aalt Bast, Pieter A. Leermakers, Christy B. M. Tulen, Paul M. H. Schiffers, Frederik J. van Schooten, Alex H. V. Remels

**Affiliations:** 1 School of Nutrition and Translational Research in Metabolism (NUTRIM), Department of Pharmacology and Toxicology, Maastricht University Medical Center+, Maastricht, The Netherlands; 2 School for Cardiovascular Diseases (CARIM), Department of Internal Medicine, Maastricht University Medical Center+, Maastricht, The Netherlands; 3 School for Oncology and Developmental Biology (GROW), Department of Obstetrics and Gynaecology, Maastricht University Medical Center+, Maastricht, The Netherlands; 4 School for Cardiovascular Diseases (CARIM), Department of Pharmacology and Toxicology, Maastricht University Medical Center+, Maastricht, The Netherlands

**Keywords:** Preeclampsia, Placenta, Mitochondria, Oxidative stress, Mitophagy, Glycolysis

## Abstract

Preeclampsia complicates 5–8% of all pregnancies worldwide, and although its pathophysiology remains obscure, placental oxidative stress and mitochondrial abnormalities are considered to play a key role. Mitochondrial abnormalities in preeclamptic placentae have been described, but the extent to which mitochondrial content and the molecular pathways controlling this (mitochondrial biogenesis and mitophagy) are affected in preeclamptic placentae is unknown. Therefore, in preeclamptic (*n* = 12) and control (*n* = 11) placentae, we comprehensively assessed multiple indices of placental antioxidant status, mitochondrial content, mitochondrial biogenesis, mitophagy, and mitochondrial fusion and fission. In addition, we also explored gene expression profiles related to inflammation and apoptosis. Preeclamptic placentae were characterized by higher levels of oxidized glutathione, a higher total antioxidant capacity, and higher mRNA levels of the mitochondrial-located antioxidant enzyme manganese-dependent superoxide dismutase 2 compared to controls. Furthermore, mitochondrial content was significantly lower in preeclamptic placentae, which was accompanied by an increased abundance of key constituents of glycolysis. Moreover, mRNA and protein levels of key molecules involved in the regulation of mitochondrial biogenesis were lower in preeclamptic placentae, while the abundance of constituents of the mitophagy, autophagy, and mitochondrial fission machinery was higher compared to controls. In addition, we found evidence for activation of apoptosis and inflammation in preeclamptic placentae. This study is the first to comprehensively demonstrate abnormalities at the level of the mitochondrion and the molecular pathways controlling mitochondrial content/function in preeclamptic placentae. These aberrations may well contribute to the pathophysiology of preeclampsia by upregulating placental inflammation, oxidative stress, and apoptosis.

**Supplementary Information:**

The online version contains supplementary material available at 10.1007/s43032-021-00464-y.

**Figure:**
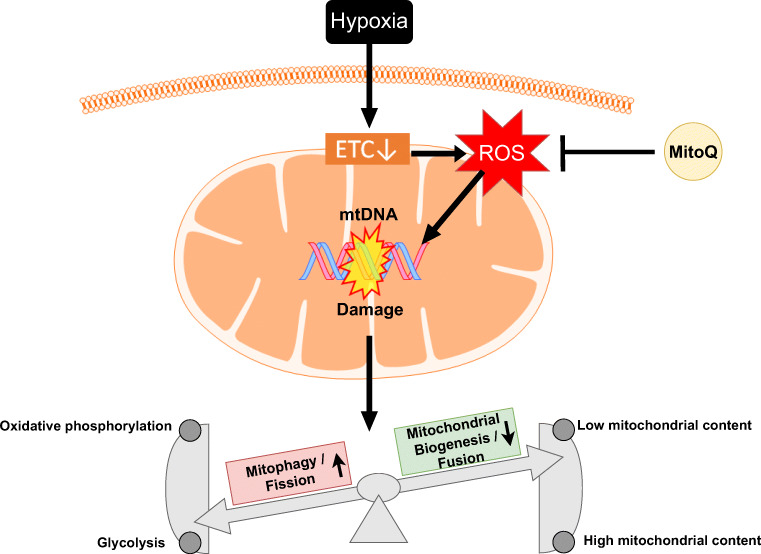
Graphical Abstract

## Introduction

Preeclampsia (PE) is a pregnancy disorder that occurs in 5–8% of all pregnancies and has potentially devastating consequences for both mother and fetus. The clinical spectrum of PE ranges from relatively mild to life-threatening, and PE is estimated to be responsible for 70,000–80,000 maternal deaths and 500,000 perinatal deaths worldwide every year [1]. PE is not simply de novo onset of hypertension and proteinuria after 20 weeks of gestation, but rather a syndrome involving multiple organs resulting in end-organ damage in terms of cardiovascular, respiratory, central nervous, renal, and hepatic systems [2, 3]. Pre-term delivery is often the only definite treatment for PE, which is associated with adverse short- and long-term health outcomes in the offspring including a high prevalence of subsequent endocrine and metabolic diseases in children [4, 5]. The pathophysiology of PE remains enigmatic, and except for delivery, no curative treatment currently exists. There is substantial evidence that poor invasion of extra-villous trophoblasts into the uterine wall and spiral arteries leads to a perturbed utero-placental circulation. Although the underlying cellular and molecular mechanisms involved remain unknown, it is believed that, as pregnancy progresses, this aberrant placental perfusion induces further damage and elicits oxidative stress in the placenta, which contributes to the ongoing development of the disease [6–8].

It is well-known that disturbed placental perfusion, as observed in patients suffering from PE, can trigger the production of reactive oxygen species (ROS) [9, 10]. Cellular responses in the placenta to ROS and ROS-induced damage in PE include the activation/upregulation of several antioxidant systems (e.g., superoxide dismutase (SOD) and catalase), as well as apoptosis of trophoblasts [11]. Importantly, oxidative stress does not only trigger trophoblast cell death but also accelerates trophoblast turnover and its secretome into the maternal circulation. These changes in the trophoblast secretome include an increased release of vasoactive factors activating the angiotensin I and endothelin-1 receptor as well as an enhanced release of inflammatory cytokines and chemokines [2, 12–14]. Alterations in secretion of these placental factors into the maternal circulation induce inflammation, endothelial dysfunction, and PE-like symptoms including hypertension [2]. Although the presence of placental oxidative stress and its contribution to the PE pathophysiology are commonly acknowledged, the sources of ROS and the exact underlying processes of its formation remain obscure. Clinical trials using vitamins C and E for the treatment of PE have been unsuccessful, illustrating that systemically acting antioxidants to restore redox imbalance have no clinical relevance [15]. Similar to observations in other clinical fields, research is directed nowadays into finding more specific redox targets with the purpose of combating oxidative stress in a more selective way, as for instance in the field of respiratory diseases [16].

Recently, mitochondria, a main intracellular source of ROS, have gained more interest as a potential novel therapeutic target in PE. In a recent study, administration of MitoQ, a mitochondrially targeted antioxidant, improved fetal outcomes, including birth weight and developmental programming of cardiovascular diseases, in a rodent model (chronic placental hypoxia) for PE [17–20]. However, whether mitochondrial dysfunction is present in PE placentae and how this contributes to the development of oxidative stress and the pathophysiology of this disease is still unknown.

Therefore, in this study, using PE and control placentae, we comprehensively assessed not only the presence of oxidative stress and the status of well-known cellular antioxidant systems but also investigated, in detail, multiple indices of mitochondrial health. This included key constituents of metabolic pathways including oxidative phosphorylation, fatty acid β-oxidation, and glycolysis. Furthermore, to portray molecular mechanisms that control mitochondrial content and function, we also assessed key regulators of mitochondrial biogenesis, as well as constituents of the mitophagy/autophagy, and mitochondrial fusion and fission machinery.

## Materials and Methods

### Clinical Subject Characteristics

Human term placentae (*n* = 11) and PE-complicated placentae (*n* = 12) were collected from in total 23 women by the Department of Obstetrics and Gynecology at the Maastricht University Medical Center+. PE was diagnosed based on the International Society for the Study of Hypertension in Pregnancy (ISSHP) criteria as de novo hypertension with a systolic blood pressure ≥ 140 mmHg and/or diastolic blood pressure ≥ 90 mmHg in 2 repeated measurements (at least 6 h apart) and the co-occurrence of proteinuria (≥0.3 g/24 h or ≥ 2+ on dipstick analysis) occurring >20 weeks of gestation in previously normotensive women or when proteinuria developed >20 weeks of gestation in women with pre-existing hypertension. Exclusion criteria for control pregnancies were hypertension (pre-existing or onset during pregnancy) and/or proteinuria, also as defined by ISSHP, chromosomal anomalies, multiple gestation, and autoimmune diseases. Intrauterine growth restriction (IUGR) was defined as neonatal birthweight below the 10th centile according to Dutch perinatal registry curves. Clinical subject characteristics are presented in Table 1.

**Table 1:** Clinical subject characteristics

	Control (*n* = 11)	PE (*n* = 12)	Significance
Gestational age at delivery (weeks)	39 ± 1	33 ± 3	***
Maternal age (years)	30 ± 4	30 ± 5	Ns
Maternal BMI (kg/m^2^)	25 ± 6	24 ± 4	Ns
Neonatal birth weight (kg)	3.4 ± 0.4	2.4 ± 0.9	**
Neonatal sex (male, %)	64	58	–
IUGR (%)	0	50	–
Mode of delivery (CS, %)	55	58	–

Data presented as mean ± standard deviation. *BMI* body mass index, *IUGR* intrauterine growth restriction, *CS* cesarean section, *Ns* non-significant. Ns: *p* > 0.05, **p ≤ 0.01, ***p ≤ 0.001

### Tissue Collection

Placental biopsies (<1 cm^2^) were collected immediately (within 15 min) after delivery from the paracentral region of the placenta at the maternal side. Infarction and calcified regions were avoided. The basal plate of the specimen was removed, and the remaining tissue was rinsed in a HEPES solution (NaCl 143.3 mM, KCl 4.7 mM, MgSO_4_ 1.2 mM, KH_2_PO_4_ 1.2 mM, CaCl_2_ 2.5 mM, glucose 5.5 mM, and HEPES 15 mM), snap-frozen in liquid nitrogen, crushed by a mortar while frozen, and stored at −80 °C until use.

### Preparation of the Lysates

Approximately 40 mg of powdered placental tissue was homogenized for 10 s at maximal speed with a handheld PRO Scientific Bio-Gen PRO200 homogenizer in 800 μl KPE lysis buffer (13 mM KH_2_PO_4_, 68 mM K_2_HPO_4_, 9 mM EDTA, and 1% Triton X-100) for antioxidant enzyme activity assays or in 800 μl SET buffer (250 mM sucrose, 2 mM EDTA, 10 mM Tris, pH 7.4) for metabolic enzyme activity assays. KPE homogenates were subsequently centrifuged at 20,000×*g* for 10 min at 4 °C. Subsequently, lysates (300 μl) were stored at −80 °C for assessment of Trolox equivalent antioxidant capacity (TEAC). The remaining lysate was mixed with 2.6% bovine serum albumin (BSA) (1:1) and stored at −80 °C for determination of glutathione/glutathione disulfide (GSH/GSSG) levels. SET homogenates were snap-frozen in liquid nitrogen, defrosted, incubated on ice for 30 min, and subsequently centrifuged at 20,000×*g* for 2 min at 4 °C. Five percent BSA was added to the lysate (1:4) and stored at −80 °C for the assessment of citrate synthase (CS), β-hydroxyacyl-CoA dehydrogenase (HADH) and phosphofructokinase (PFK) activity. For DNA and RNA isolation, 40 mg of powdered placental tissue was homogenized in 1 ml Trizol reagent (Invitrogen Corp.) and further processed according to the manufacturer’s protocol (Catalog Number 15596026 and 15596018, Invitrogen™, USA). For generation of whole cell lysates (for western blot analysis), 40 mg of powdered placental tissue was homogenized in 800 μl IP lysis buffer (50 mM Tris, 150 mM NaCl, 10% glycerol, 0.5% Nonidet P40, 1 mM EDTA, 1 mM Na_3_VO_4_, 5 mM NaF, 10 mM β-glycerophosphate, 1 mM Na_4_O_7_P_2_, 1 mM DTT, 10 μg/μl leupeptin, 1% apropeptin, 1 mM PMSF, pH 7.4) using the homogenizer for 20 s at maximal speed. Lysates were incubated for 30 min on ice and centrifuged at 20,000×*g* for 30 min at 4 °C. Lysates were aliquoted (1 μg/μl) in Laemmli buffer buffer (0.25 M Tris-HCl, 8% (w/v) SDS, 40% (v/v) glycerol, 0.4 M DTT, 0.04% (w/v) bromophenol blue, pH 6.8) and boiled for 5 min at 95 °C. Protein concentrations of whole cell lysates and enzyme activity lysates were determined using the Pierce™ BCA Protein Assay kit according to the manufacturer’s protocol (Pierce Chemical Co., Rockford, IL). For the HADH, PFK, CS, TEAC, and GSSH/GSH assay, the number was lower (9 controls and 8 PE placentae and not 11 controls and 12 PE placentae as used for the western blot and qPCR measurements), because there was not sufficient tissue.

### Glutathione Disulfide/Glutathione Levels

The GSH assay was performed for the determination of the levels of GSH + GSSG and GSSG as described previously [21]. First, GSH (0.1–10 μM) and GSSG (0.1–5 μM) standards were prepared in a KPE buffer and 1.3% 5-sulfosalicylic acid. GSSG standards and samples were diluted 1:10 with 2-vinylpyridine, incubated, and mixed for 1 h to form a stable complex with GSH, preventing it from participating in the enzymatic recycling reaction with glutathione reductase. Reactions were set up in a 96-well plate, and 50 μl of sample was loaded in duplicate. Reactions were initiated by adding 100 μl 0.8 mM NADPH/0.6 mM DTNB 1:1 and 4 U/ml GSSG reductase to the samples. Color development of samples and standards was recorded kinetically for 3 min in 9 reads at 412 nM resulting in GSH + GSSG and GSH slope values. Activity was corrected for total protein content of the samples and expressed in nM/mg protein/min.

### Trolox Equivalent Antioxidant Capacity

The Trolox equivalent antioxidant capacity (TEAC value) is a measurement for total antioxidant status, relating the free radical scavenging properties of a solution or a compound to that of the synthetic antioxidant Trolox, and was performed as described earlier [22]. First, a 5 mM 2,2′-azino-bis(3-ethylbenzothiazoline-6-sulphonic acid) (ABTS) solution was prepared in 145 mM sodium phosphate buffer (pH 7.4). Then, an (ABTS^•−^) solution was prepared by adding 10 μl 1/100 horseradish peroxidase (HRP) and 10 μl of 2 mM H_2_O_2_ solution and diluted in an ABTS solution to a final absorbance of 0.70 ± 0.02 at 734 nm at 37 °C. Deproteinization of samples was performed by adding 10% trichloroacetic acid (TCA) (1:1) to the samples. For measuring antioxidant capacity, 50 μl of the lysate was mixed with 950 μl ABTS^•−^ solution in duplicate at 37 °C for 5 min, and absorbance was measured at 734 nm and compared to the absorbance of an ABTS^•−^ solution without sample. Absorbance was corrected for total protein content.

### Citrate Synthase Activity

As previously described (CS; EC 2.3.3.1) [23], a reaction mix was set up in a 96-well plate in duplicate containing 5 μl undiluted sample, 200 μl reagent containing Tris (100 mM), DTNB (0.1 mM), and acetyl-coenzym A (0.3 mM). The reaction was started with 5 μl start reagent containing oxaloacetic acid (25 mM). Enzyme activity was monitored at 412 nm (37 °C) and corrected for total protein content.

### β-Hydroxyacyl-CoA Dehydrogenase Activity

As previously described (HADH; EC 1.1.1.35) [24], a reaction mix was set up in a 96-well plate in duplicate containing 10 μl undiluted sample, 100 μl reagent containing NADH (1.1 mM), and tetrapotassium pyrophosphate (100 mM). The reaction was started with 10 μl acetoacetyl-CoA (2.4 mM). Enzyme activity was kinetically monitored at 340 nm (37 °C) and corrected for total protein content.

### Phosphofructokinase Activity

As previously described ((PFK, EC 2.7.1.11) [25], a reaction mix was set up in a 96-well plate in duplicate containing 20 μl undiluted sample, 100 μl reagent containing Tris Base (49.6 mM), MgCl_2_.6H_2_O (7.4 mM), KCl (3.2 mM), KCN (384.6 μM), ATP (3.0 mM), DTT (1.5 mM), NADH (0.3 mM), aldolase (0.019 U), glycerol-3-phosphate dehydrogenase (0.019 U), and triose phosphate isomerase (0.019 U), pH 8.0. The reaction was started with fructose-6-phosphate (35.9 mM) in Tris buffer (49.6 mM), pH 8.0. Enzyme activity was monitored at 340 nm (37 °C) and corrected for total protein content.

### Quantitative Real-Time PCR

4.4 μl of 1:50 diluted cDNA was used for quantitative PCR amplification using target specific primers (Supplementary Table 1) and 2X Sensimix™ SYBER® & Fluorescein mix (Bioline, Alphen aan de Rijn, the Netherlands) on a LightCycler480 384-wells PCR machine (Roche, Almere, the Netherlands). Specificity of PCR amplification was checked by melt curve analysis. Expression levels of genes of interest were corrected using a normalization factor calculated based on the expression of 2 different housekeeping genes (Cyclophilin A and Ribosomal Protein L13a (*RPL13A*)), which were found to be most stable from a selection of 3 genes by using the GeNorm software (Primerdesign, Southampton, USA). The list of primers can be found in Supplementary Table 1.

### Mitochondrial DNA Copy Number

4.4 μl of 1:25 diluted DNA was used for qPCR as described above, using mitochondrial DNA (mtDNA, cytochrome C oxidase subunit 2 (*COXII*)) and genomic DNA (gDNA, *RPL13A*)—specific primers (Supplementary Table 1). mtDNA/gDNA ratio was determined by dividing the relative quantity of mtDNA by the relative quantity of gDNA.

### Western Blotting

Ten micrograms of protein was run through a Criterion XT 4–12 or 12% Bis-Tris gel (Bio-Rad, Veenendaal, the Netherlands) in 1x MES running buffer (Bio-Rad, Veenendaal, the Netherlands) at 100 V and was subsequently blotted on a nitrocellulose membrane (Bio-Rad Laboratories B.V., Veenendaal, the Netherlands) by electroblotting. At least two protein ladders were loaded on each gel (Precision Plus Protein™ All Blue Standards #161-0373, Bio-Rad Laboratories, Veenendaal, the Netherlands). Membranes were stained with 0.2% Ponceau S in 1% acetic acid (Sigma-Aldrich, Zwijndrecht, the Netherlands) for 5 min, washed with Milli-Q, and imaged using the Amersham™ Imager 600 (GE Healthcare, Eindhoven, the Netherlands) to quantify total protein content as correction for gel-loading. Membranes were blocked for 1 h with Tween20 Tris-buffered saline (TBST; 20 mM Tris, 137 mM NaCl, 0.1% (vol/vol) Tween20, pH 7.6) containing 3% (w/v) non-fat dry milk (Campina, Eindhoven, the Netherlands), washed, and incubated overnight at 4 °C with a target-specific primary antibody (S2 Table) diluted 1:1000–1:10,000 in TBST with 3% (w/v) BSA or non-fat dry milk at 4 °C. Subsequently, membranes were washed and incubated with a HRP-conjugated secondary antibody (#BA-9200, #BA-1000, Vector Laboratories, Amsterdam, the Netherlands), diluted 1:10,000 in 3% (w/v) non-fat dry milk in TBST for 1 h at room temperature. Thereafter, membranes were washed, incubated for 5 min with 0.5x SuperSignal West PICO or 0.25x West Femto Chemiluminescent Substrate (Thermo Scientific, Landsmeer, the Netherlands), and imaged using the Amersham™ Imager 600. Original unaltered images were quantified with Image Quant software (GE Healthcare, Eindhoven, the Netherlands). Measured protein quantity was corrected for total protein content. Images included in the figures of this manuscript have been adjusted for brightness and contrast equally throughout the picture. Antibodies used for western blot can be found back in Supplementary Table 2.

### Statistical Analysis

Data is depicted as bar graphs indicating the mean and SEM as fold change compared to the control. For each comparison, the D’Agostino and Pearson omnibus normality test was used to test normality, and subsequently either an unpaired *t* test or Mann-Whitney test was used accordingly (GraphPad Software, La Jolla, CA, USA). The SPSS statistical software (IBM Corp. Released 2016. IBM SPSS Statistics for Windows, Version 24.0. Armonk, NY: IBM Corp) was used to perform a regression. A *p* value < 0.05 was considered significantly different from the control group and was presented as follows: Ns: *p* > 0.05, *p ≤ 0.05, **p ≤ 0.01, and ***p ≤ 0.001.

## Results

### Increased Oxidative Stress in PE Placentae

**Fig. 1:**
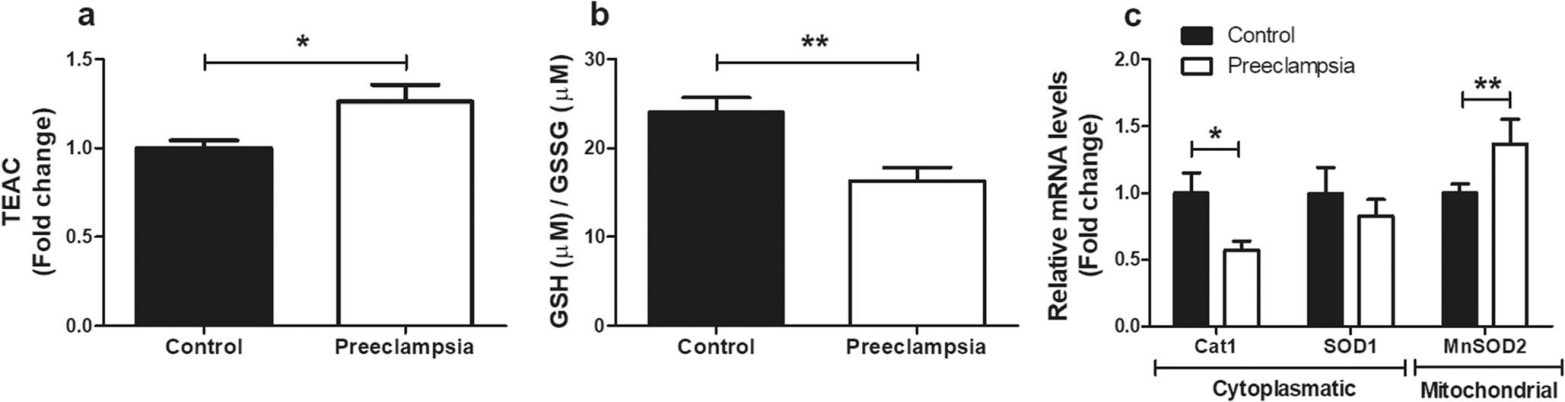
Oxidative stress in PE placentae. TEAC (**a**) and GSH/GSSG ratio (**b**) as well as transcript levels of *CAT1* and *CuZnSOD1* and *MnSOD2* (**c**) were assessed in PE as well as control placentae. Data is presented as fold change compared to the control placentae and as mean with SEM from *n* = 9 (controls), *n* = 8 (preeclampsia) for assessment of TEAC and GSH/GSSG ratio and *n* = 11 (controls), *n* = 12 (preeclampsia) for assessment of mRNA levels. *p ≤ 0.05, **p ≤ 0.01. TEAC, Trolox equivalent antioxidant capacity; GSSG, oxidized glutathione; GSH, reduced glutathione; Cat1, catalase-1; SOD1, superoxide dismutase 1; and MnSOD2, manganese-dependent superoxide dismutase

To determine the impact of PE on known ROS-generating processes and on cellular antioxidant defense systems, mRNA abundance and enzymatic activity of key constituents of these pathways were assessed. As shown in Fig. 1, TEAC and the GSH/GSSG ratio were significantly higher in PE placentae compared to controls (Fig. 1a–b). In addition, in PE placentae, transcript levels of catalase-1 (*CAT1*) were lower, while mRNA expression levels of *CuZnSOD1* and mitochondrial manganese-dependent superoxide dismutase 2 (*MnSOD2*) were respectively unchanged and significantly higher compared to controls (Fig. 1c). No differences were observed for transcript abundance of xanthine oxidase (*XO*), NADPH oxidase 2 (*NOX*2), or *NOX4* (Supplementary Fig. 1a). In addition to these indicators of oxidative stress in PE placentae, both the expression of tumor necrosis factor-α (*TNF-α*) and the ratio of pro-apoptotic Bcl-2-associated X protein/anti-apoptotic B cell lymphoma 2 (*BAX*/*BCL-2*) mRNA expression levels were significantly higher in PE placentae compared to controls (Supplementary Fig. 1b-c).

### Lower Mitochondrial Content but Increased Glycolysis in Placentae of PE Patients Compared to Controls

**Fig. 2:**
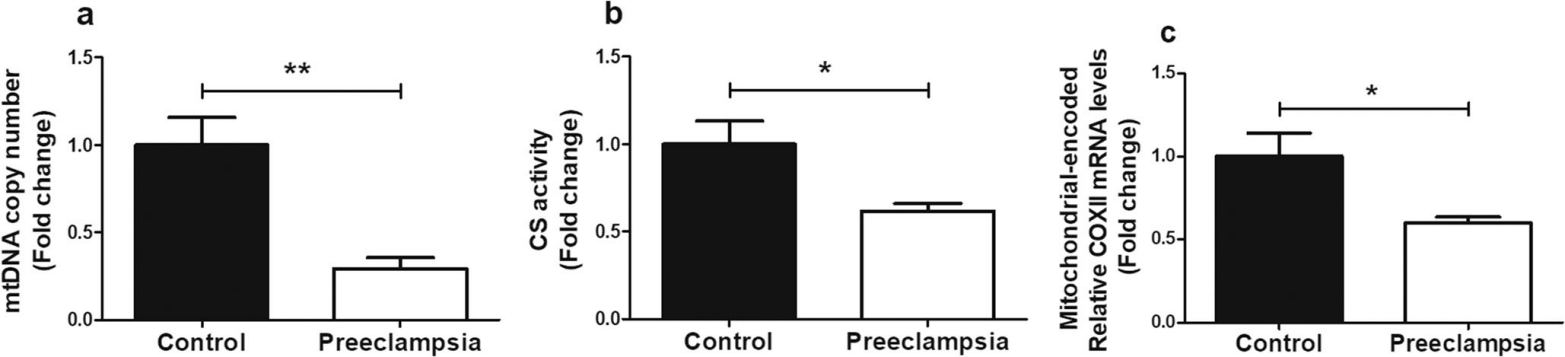
Lower mitochondrial content in PE placentae. Mitochondrial DNA copy number (**a**), citrate synthase activity (**b**), and mRNA transcript levels of COXII (**c**) were assessed in PE as well as control placentae. Data is presented as fold change compared to the control placentae and as mean with SEM from *n* = 11 (controls), *n* = 12 (preeclampsia) for assessment of mRNA levels and *n* = 9 (controls), *n* = 8 (preeclampsia) for the assessment of CS activity and mtDNA copy number. *p ≤ 0.05 and **p ≤ 0.01. mtDNA, mitochondrial DNA; CS, citrate synthase; COXII, cytochrome *c* oxidase subunit II

**Fig. 3:**
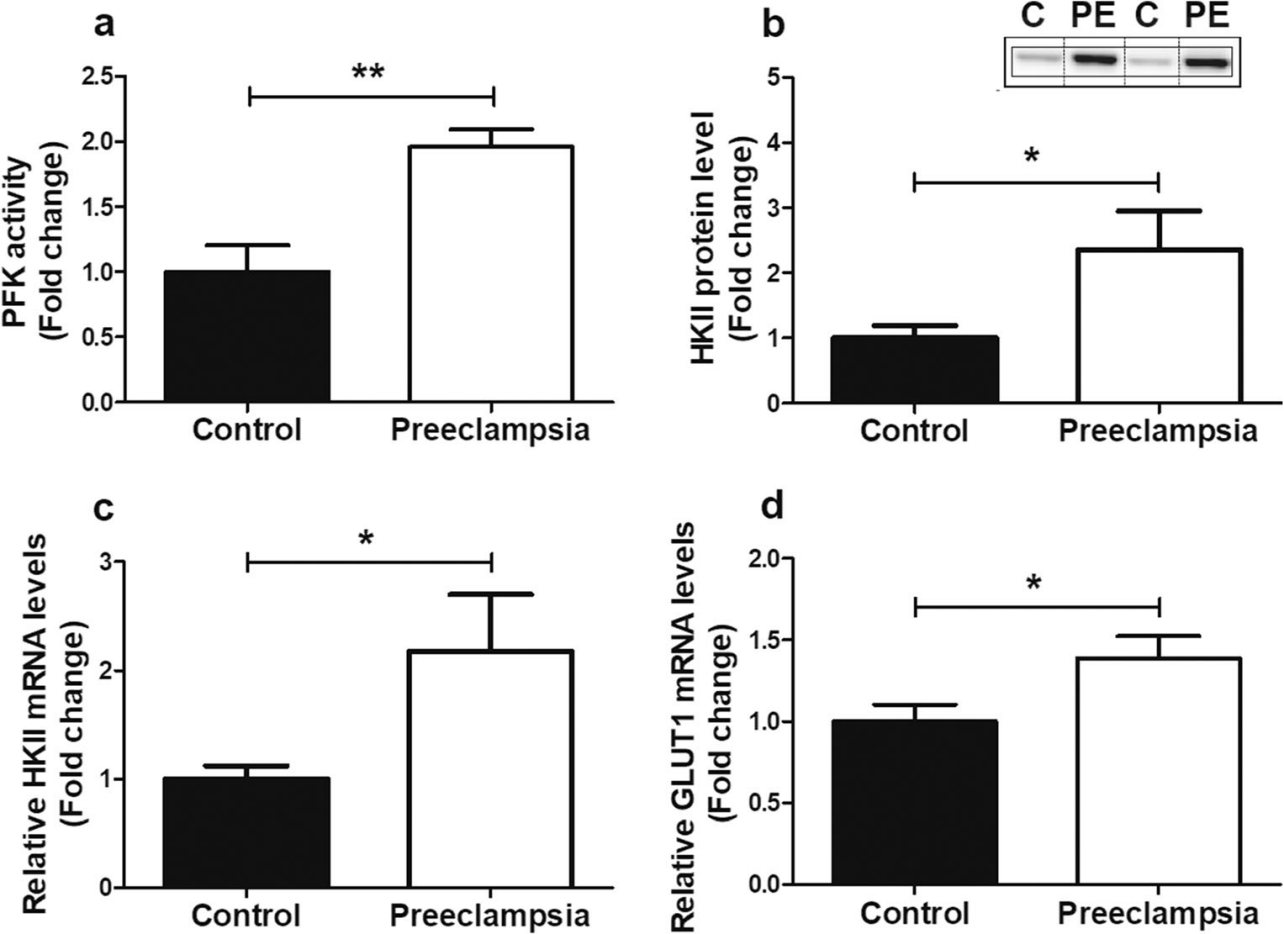
Increased expression and activity of key glycolytic enzymes in PE placentae. PFK enzyme activity (**a**), HKII protein level (**b**), mRNA expression levels of *HKII* (**c**), and *Glut-1* (**d**) were assessed in PE as well as control placentae. Representative immunoblots are shown, and western blots were corrected for total protein loading assessed by Ponceau S staining with adjusted contrast equally applied to the whole photograph. Black boxes around the representative pictures indicate that they were cut from the same western blot. Data is presented as fold change compared to the control placentae and as mean with SEM from *n* = 11 (controls), *n* = 12 (preeclampsia) for assessment of mRNA levels and *n* = 9 (C: controls), *n* = 8 (PE: preeclampsia) for assessment of the PFK activity and HKII protein levels. Ns: *p* > 0.05, *p ≤ 0.05, **p ≤ 0.01. PFK, phosphofructokinase; HKII, hexokinase; and GLUT-1, glucose transporter 1

As mitochondria are known drivers of apoptosis and inflammation [26] and are a well-known source of ROS, and ROS are known to be able to damage mitochondria and impair mitochondrial function [26], we next investigated whether PE affected placental mitochondrial content and mitochondrial (and non-mitochondrial) metabolic processes. Mitochondrial content was significantly lower in PE placentae compared to controls, which was evidenced by a lower mitochondrial DNA (mtDNA) copy number (Fig. 2a) and lower total enzyme activity of CS (Fig. 2b), two well-described indicators of mitochondrial content [27]. No changes were observed in mRNA transcript levels of *CS* (Supplementary Fig. 2a). To investigate if the lower mitochondrial content that we observed in PE placentae was associated with changes in (mitochondrial and non-mitochondrial) metabolic processes, we next assessed the abundance and activity of key constituents of the electron transport chain (ETC), fatty acid-β oxidation (FAO), and glycolysis. Interestingly, while no differences were found in activity or mRNA expression levels of the rate-limiting enzyme of the FAO pathway (Supplementary Fig. 2b-c) or in transcript and protein abundance of nuclear-encoded sub-units of ETC complexes (Supplementary Fig. 2d-e), we did observe that mRNA transcript levels of the mitochondrial-encoded sub-unit *COXII* of complex IV of the ETC were significantly lower in PE placentae compared to controls (Fig. 2c). Moreover, the PFK activity as well as protein and mRNA abundance of hexokinase II (HKII), two key enzymes involved in glycolysis, were 2–3-fold higher in PE placentae compared to controls (Fig. 3a–c). This was also associated with higher transcript levels of the glucose transporter 1 (GLUT-1) in PE placentae (Fig. 3d).

### Alterations in the Molecular Regulation of Mitochondrial Biogenesis in PE Placentae

**Fig. 4:**
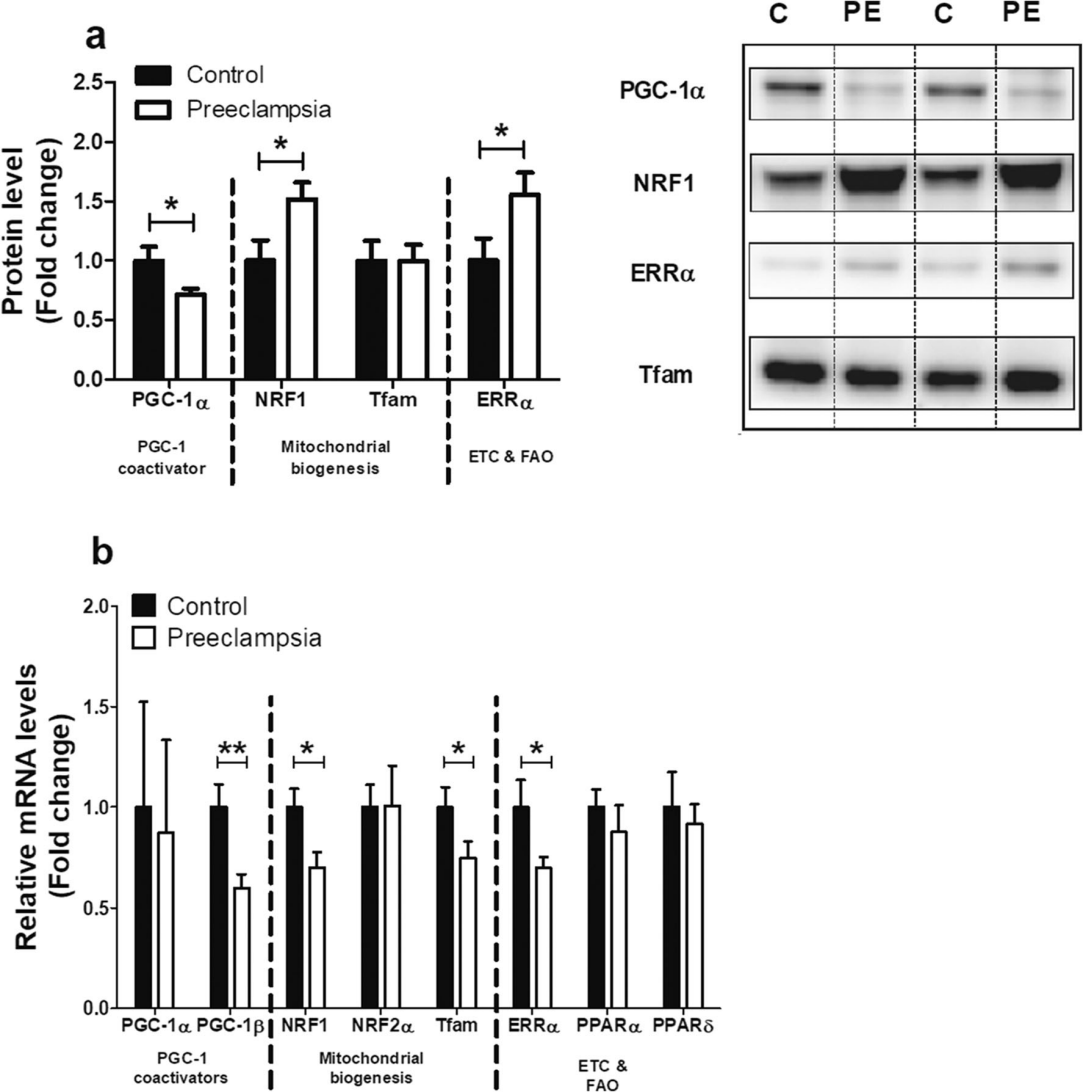
Alterations in the molecular regulation of mitochondrial biogenesis in PE placentae. Protein levels of PGC-1α, NRF1, Tfam, and ERRα (**a**) and mRNA transcript levels of *PGC-1α*, *PGC-1β*, *NRF1*, NRF2α, *Tfam, ERRα, PPARα*m and *PPARδ* (**b**) were assessed in PE as well as control placentae. Representative immunoblots are shown, and western blots were corrected for total protein loading assessed by Ponceau S staining with adjusted contrast equally applied to the whole photograph. Black boxes around the representative pictures indicate that they were cut from the same western blot. Data is presented as fold change compared to the control placentae and as mean with SEM from *n* = 11 (controls), *n* = 12 (preeclampsia). *p ≤ 0.05, **p ≤ 0.01. PGC-1α, peroxisome proliferator-activated receptor gamma coactivator 1-alpha; NRF1, nuclear respiratory factor 1; Tfam, mitochondrial transcription factor A; ERRα, estrogen-related receptor alpha; PGC-1β, peroxisome proliferator-activated receptor gamma coactivator 1-beta; NRF2α, nuclear respiratory factor 2 alpha; PPARα, peroxisome proliferator-activated receptor alpha; PPARδ, peroxisome proliferator-activated receptor delta; ETC, electron transport chain; and FAO, fatty acid β-oxidation

In light of the reductions in mitochondrial content and indications for a metabolic shift away from oxidative metabolism in PE placentae, we next explored whether PE impacts the peroxisome proliferator-activated receptor gamma coactivator 1 (PGC-1) signaling network, a key regulatory network controlling mitochondrial biogenesis and mitochondrial oxidative substrate metabolism through the coordinated action of a variety of transcription factors and co-activator molecules [28]. As depicted in Fig. 4, while protein levels of PGC-1α were significantly lower in PE placentae, no changes in *PGC-1α* transcript levels were observed compared to controls. Placental mRNA levels of *PGC-1β*, however, were significantly lower in PE. With regard to transcription factors specifically controlling mitochondrial biogenesis, protein levels of nuclear respiratory factor 1 (NRF1) were higher, and mRNA expression of *NRF1* was lower in PE placentae vs controls. Placental *NRF2α* mRNA levels on the other hand were not affected by PE. Additionally, although mitochondrial transcription factor A (*Tfam*) transcript abundance was lower in PE, no significant differences in Tfam protein levels were observed between both groups. Furthermore, estrogen-related receptor alpha (ERRα) protein levels were higher in PE, while its mRNA levels were lower. Transcript abundance of both peroxisome proliferator-activated receptor (*PPAR*)*-α* and *PPAR-δ* showed no significant differences between both groups (Fig. 4a–b). Collectively, these data indicate significant alterations in the molecular regulation of mitochondrial biogenesis and oxidative substrate metabolism in PE compared to controls.

### Expression of Key Constituents of the Mitophagy Machinery Are Altered in PE Placentae

**Fig. 5:**
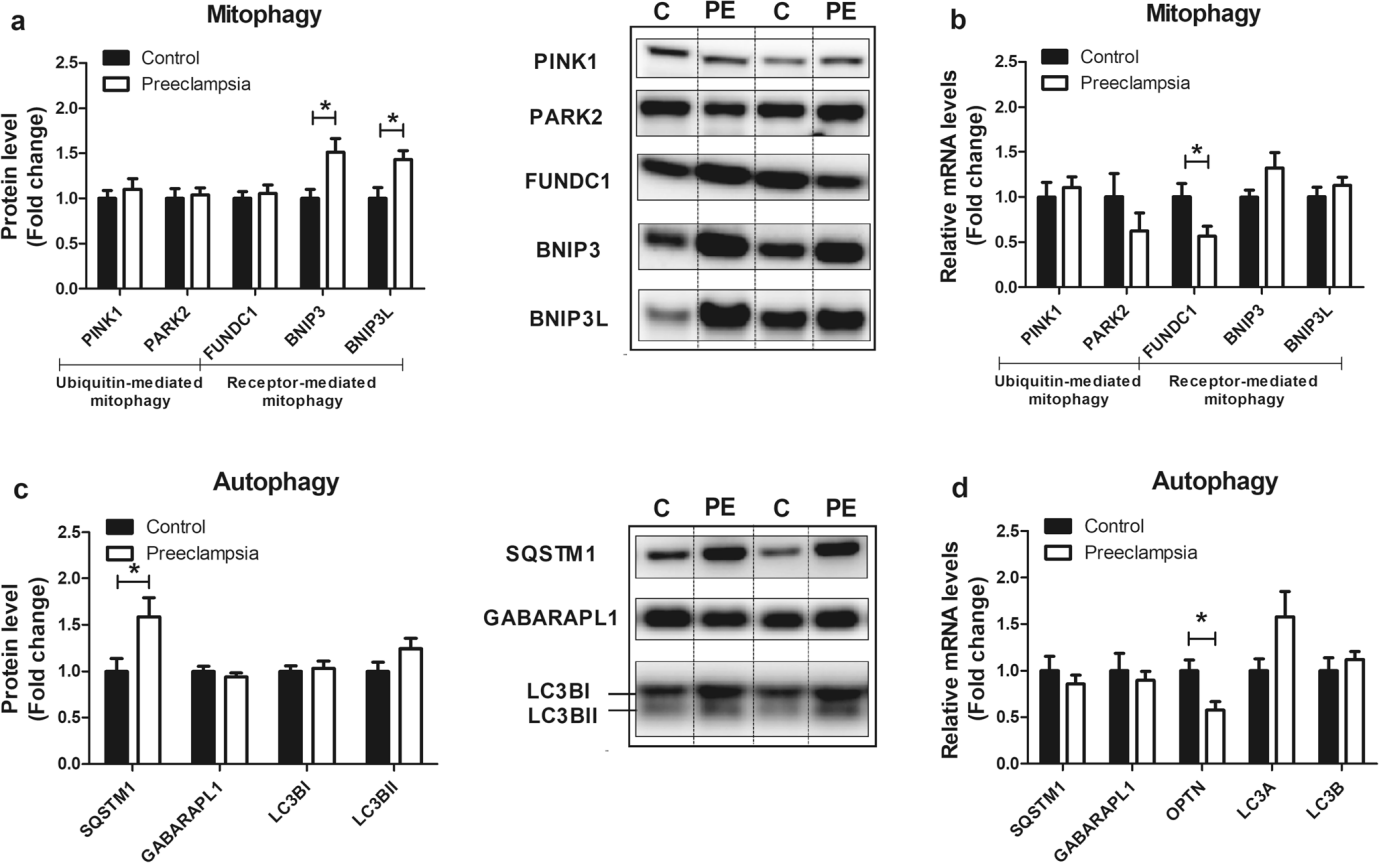
Expression of key constituents of the mitophagy machinery are altered in PE placentae. Mitophagy-associated protein levels of PINK1, PARK2, FUNDC1, BNIP3, and BNIP3L (**a**); mitophagy-associated mRNA transcript levels of *PINK1*, *PARK2*, *FUNDC1*, *BNIP3*, and *BNIP3L* (**b**); autophagy-associated protein levels of SQSTM1, GABARAPL1, LC3BI, and LC3BII (**c**); and autophagy-associated mRNA transcript levels of *SQSTM1*, *GABARAPL1*, *OPTN*, and *LC3A/B* (**d**) were assessed in PE as well as control placentae. Representative immunoblots are shown and western blots were corrected for total protein loading assessed by Ponceau S staining with adjusted contrast equally applied to the whole photograph. Black boxes around the representative pictures indicate that they were cut from the same western blot. Data is presented as fold change compared to the control placentae and as mean with SEM from *n* = 11 (controls), *n* = 12 (preeclampsia). *p ≤ 0.05. PINK1, PTEN-induced kinase 1; PARK2, E3 ubiquitin-protein ligase Parkin; FUNDC1, FUN14 domain containing 1; BNIP3, BCL2/adenovirus E1B 19 kDa protein-interacting protein 3; BNIP3L, BCL2/adenovirus E1B 19 kDa protein-interacting protein 3-like; SQSTM1, Sequestosome 1; GABARAPL1, GABA Type A Receptor Associated Protein Like 1; LC3B, microtubule-associated protein 1 light chain 3 beta I/II; OPTN, optineurin; and LC3A, microtubule-associated protein 1 light chain 3 alpha

As mitochondrial content is not only controlled by the biogenesis of new organelles but is also influenced by breakdown of mitochondria through mitophagy (e.g., mitochondrial-specific autophagy), we next assessed the impact of PE on key constituents of the mitophagy machinery. Moreover, as mitophagy requires several general autophagy-related proteins for generating the autophagosomal membrane and priming the autophagosome to the mitochondria, these proteins were studied as well. As depicted in Fig. 5a, in PE placentae, protein levels of BCL2/adenovirus E1B 19 kDa protein-interacting protein 3 (BNIP3) and BCL2/adenovirus E1B 19 kDa protein-interacting protein 3-like (BNIP3L) were significantly higher, while abundance of FUN14 domain containing 1 (FUNDC1), the PTEN-induced kinase 1 (PINK1), and E3 ubiquitin-protein ligase Parkin (PARK2) proteins were similar compared to controls. mRNA expression levels of these mitophagy-associated proteins were unaltered in PE placentae compared to the controls with the exception of *FUNDC1* transcript levels, which were lower in PE (Fig. 5b). In addition, protein and mRNA transcript levels of all general autophagy-associated proteins investigated were not significantly different in PE vs controls. Sequestosome 1 (SQSTM1) protein levels, however, were significantly higher, and mRNA transcript levels of optineurin (*OPTN*) were significantly lower in PE placentae compared to controls (Fig. 5c–d). Collectively, these data indicate that proteins specifically involved in receptor-mediated mitophagy (BNIP3, BNIP3L), rather than ubiquitin-mediated mitophagy (PINK1, PARK2), are higher in PE placentae compared to controls.

### Increased Abundance of Mitochondrial Fission Proteins in PE Placentae

As mitochondrial fission and fusion are key events in the processes of mitochondrial biogenesis as well as mitophagy and are essential in maintaining normal mitochondrial homeostasis [29], mRNA and protein levels of several mitochondrial fusion and fission proteins were investigated. Protein levels of dynamin-1-like protein (DNM1L), a key protein involved in mitochondrial fission, were significantly higher in PE placentae (Supplementary Fig. 3a). Furthermore, mitochondrial fission-related mRNA transcript levels of *DNM1L* were also significantly higher in PE, while fission 1 (*Fis-1*) transcript levels were unaltered compared to controls (Supplementary Fig. 3b). With regard to mediators of mitochondrial fusion, mRNA transcript levels of mitofusin-1 and 2 (*Mfn1* and *Mfn2*) and mitochondrial Optic atrophy protein 1 (*Opa1*) were not significantly different in PE placentae compared to controls (Supplementary Fig. 3c), indicating that specifically mitochondrial fission constituents are higher in PE placentae compared to controls.

## Discussion

### Principal Findings

In the current study, we show that PE placentae are not only characterized by the presence of oxidative stress but also by a profound reduction in mitochondrial content and an increased abundance of key glycolytic enzymes suggestive of a greater reliance on glycolytic metabolism. Moreover, we show for the first time that these changes are associated with significant alterations in the molecular pathways governing control over mitochondrial biogenesis and changes in key constituents of the machinery controlling mitophagy and mitochondrial fission events. Taken together, our findings suggest significant alterations at the level of the mitochondrion in placentae from women with PE, which may well contribute to PE pathophysiology.

The presence of oxidative stress and associated changes in several intracellular antioxidant systems is well described in PE [10]. For example, PE has been associated with decreased activity levels of the antioxidant enzymes catalase and superoxide dismutase, increased lipid peroxidation by-products, reduced GSH production, impaired GSSG to GSH conversion, and reduced levels of non-enzymatic antioxidants such as thiols, ascorbic acid, α-tocopherol, and carotenoids in the placenta [30–32]. Collectively, these observations are in line with our data. We observed no changes in expression levels of NOX 2/4 and XO isoforms, two well-known sources of intracellular ROS. Levels of oxidized glutathione however were increased, whereas catalase mRNA expression was lower and transcript levels of the mitochondrial-located *MnSOD2* were increased in PE placenta suggestive of changes in oxidant status. This was also reflected by the fact that total antioxidant capacity in our study was markedly higher in PE placentae, suggestive of a compensatory response to increased oxidative stress in PE placentae [30, 31]. Since we only assessed mRNA expression levels (and not activity levels) of a few known antioxidants (catalase and SOD), from our data, we cannot pinpoint which antioxidant system is primarily driving this increase in total antioxidant capacity. Considering the signs for increased oxidative stress, elevated mitochondrial-specific antioxidant expression in PE placentae and the notion that the mitochondria are the main intracellular site for cellular O_2_ consumption, these data suggest that mitochondria likely are a significant source for ROS and oxidative stress in PE.

Increased placental mitochondrial ROS may directly damage mtDNA and disrupt mitochondrial metabolic function and mitochondrial biogenesis [33]. In this context, we found evidence for significant reductions in mitochondrial content in PE placentae compared to controls, which was illustrated by reductions in mtDNA copy number and reduced citrate synthase activity, both well-established markers of mitochondrial content [27]. In the placenta, disorders related to impaired perfusion including IUGR and PE have recently been shown to be associated with changes in mitochondrial content (both increased and decreased) [34]. In addition, a recent study using transmission electron microscopy revealed swelling, increased mitochondrial lumen, and irregular arrangement of mitochondrial cristae in early-onset PE placentae [32]. The majority of studies investigating mitochondrial content in PE-complicated placentae used whole placental tissue homogenates. However, mitochondria within different cell lineages in the placentae often have distinct functions and different antioxidant capacity and do respond differently to environmental stimuli [34]. Indeed, Mando et al. found increased mitochondrial content in whole placental tissue but decreased content in cytotrophoblast cells in PE, which are in direct contact with maternal blood and are therefore expected to be most affected by impaired placenta perfusion in PE [34]. This finding underlines the need for more standardized sampling methods in order to better compare mitochondrial content in placentae between different studies. Furthermore, PE placentae show increased numbers of mitochondria in cytotrophoblast cells, but with reduced size [35, 36], which is suggestive of increased mitochondrial fission. In light of these findings, it remains to be established in which cell types of the placenta mitochondrial content is affected.

Besides changes in mitochondrial content, several human studies showed a significant reduction of adenosine triphosphate (ATP) levels in PE placentae [37–39], indicating impaired functioning of the mitochondrial metabolic pathways including the ETC. In line with this, a PE rat model of reduced uterine perfusion pressure showed lower complex I and complex IV activity in the placenta [40]. Our PE placentae showed signs for a decrease in the abundance of complex III of the mitochondrial ETC. This is in line with previous work, which showed reductions in the abundance of COXII and impaired electron flow through complex IV, which these authors suggested to be contributing to excessive mitochondrial ROS formation [41, 42]. In contrast to previous studies [37, 43, 44], which in general found a decrease in the expression and activity of metabolic enzymes involved in the initial step of the β-oxidation pathway, no difference was found in the activity of HADH in our PE placentae compared to the controls. Collectively, our data, in concert with available literature, suggest abnormalities in mitochondrial content and function in PE.

In line with this notion, we show for the first time that mitochondrial abnormalities in PE placentae were associated with increases in the abundance and activity of key glycolytic enzymes, suggesting an increased relative contribution of glycolysis in PE. In accordance with these results, a significant increase in the glycolytic intermediate 2-phosphoenolpyruvate was found in severe PE-complicated placentae in humans [37]. Furthermore, increased glycolysis upon hypoxic stress, a well-known stress factor in PE, has been observed in placental trophoblast cells [45].

In line with the decreased mitochondrial content found in our PE placentae, we observed decreased mRNA and protein levels of PGC-1 co-activator molecules, which are master regulators of mitochondrial biogenesis. Moreover, mRNA expression of *NRF1* and *Tfam*, both known to be downstream of PGC-1 in the induction of mitochondrial biogenesis, was found to be decreased suggesting diminution of mitochondrial biogenesis in PE placentae. Although available evidence is scarce, other studies are in line with these results and showed that in PE placentae, where mitochondrial content was decreased, mRNA expression levels of *PGC1-α* and *NRF1* were also decreased [46, 47]. Moreover, placental tissue from a reduced uterine perfusion rat model also showed reductions in PGC-1α protein levels [48], suggesting that impaired placental perfusion may well be linked to mitochondrial adaptations in PE. However, in our study, NRF1 and ERRα protein levels were significantly higher in PE placentae compared to controls suggesting a potential compensatory cellular response to increased mitochondrial biogenesis. In this regard, although its regulatory pathways are clearly impacted, it remains to be established whether mitochondrial biogenesis is down- or upregulated in PE. More specifically, for example, our results indicate that NRF1 and ERRα protein abundance is increased in PE, while their transcript levels were significantly lower compared to controls. Although this is insufficient evidence to conclude whether activity of the NRF1 and ERRα proteins (which serves a key role in mediating mitochondrial biogenesis) is changed, one could argue that increased protein levels may be associated with increased transcriptional activity of these proteins, and the cell, therefore, as a negative feedback loop, downregulates transcription of these respective genes, resulting in lower mRNA levels.

In addition to mitochondrial biogenesis, selective autophagy of mitochondria (i.e., mitophagy) contributes to the regulation of cellular mitochondrial content. We observed higher levels of the receptor-mediated mitophagy-related proteins BNIP3 and BNIP3L as well as the general autophagy-related protein SQSTM1 in PE placentae compared to controls. Interestingly, these proteins have been shown to be upregulated upon hypoxic stress in the placenta in previous studies [49, 50]. In contrast, abundance of constituents of the Pink/Park pathway, another key regulatory pathway controlling (ubiquitin-mediated) mitophagy, was not affected by PE in our study indicating that specific mitophagy pathways may be involved in the pathogenesis of PE.

Both mitochondrial biogenesis and mitophagy require mitochondrial fusion and fission events. ROS production regulates mitochondrial fission and fusion in healthy cells, providing a mechanism that regulates mitochondrial morphology and function, which is dependent on the redox state [51]. Both mitochondrial fission and fusion are important mechanisms in the maintenance of mitochondrial health, and changes in these processes could therefore have a large impact on mitochondrial function [52]. In this context, we now show increased fission-related DNM1L protein and mRNA transcript levels in placentae complicated with PE, while the expression of mitochondrial fusion genes was unaltered. Therefore, the balance between mitochondrial fusion and fission may be tilted towards increased mitochondrial fission in PE, which can contribute to aberrant mitochondrial morphology and function in PE-complicated placentae.

Collectively, our data show extensive abnormalities at the level of the mitochondrion and the regulatory pathways controlling mitochondrial content and function in PE placentae. This suggests a large-scale mitochondrial dysfunction in PE placentae, which may well contribute to the pathogenesis of PE. Indeed, dysfunctional mitochondria are not only closely linked to excessive ROS formation but also to inflammation as well as apoptosis, both of which have been essentially implicated in the pathogenesis of PE [35, 53, 54]. Indeed, levels of many apoptotic proteins as well as in intracellular Ca^2+^ are altered in PE placentae [55, 56]. In agreement with this data, we found increased mRNA transcript levels of the *BAX*/*BCL-2* ratio in our PE placentae. Moreover, it has been shown that increased expression levels of BNIP3, as observed in our study, led to a decreased mitochondrial membrane potential and trigger cells apoptosis or autophagy by the activation of the Bax/Bak or LC-3/Beclin 1 signaling pathway [32]. In addition to a prominent role in the control of apoptosis, earlier studies found activation of the NF-κB pathway and increased abundance of pro-inflammatory cytokines in placentae and syncytiotrophoblast, respectively, in women with early PE [1, 57], which is in line with the increased pro-inflammatory mRNA transcript levels of *TNF-α* found in our PE placentae.

### Clinical Implications

PE is generally associated with placental oxidative stress, which is believed to play a key role in the pathophysiology of the disease. The underlying molecular mechanisms for the excessive production of placental ROS, however, remain unclear [10]. Considering the signs for increased oxidative stress and mitochondrial dysfunction found in our PE placenta, and the notion that the mitochondria are the main intracellular site for cellular O_2_ consumption, it is feasible that mitochondria are a significant source for the increased production of placental ROS in PE. This may open up new avenues for new treatment options for PE. Illustrative of the clinical relevance of our findings, in a rodent model for PE (chronic placental hypoxia) administration of the mitochondrial-targeted antioxidant MitoQ, improved fetal outcomes, including birth weight and developmental programming of cardiovascular diseases [17–20]. As observed in our study, increased placental mitochondrial ROS may directly damage mtDNA, inhibit mitochondrial biogenesis, and promote mitophagy. The increased reliance on glycolysis in PE placentae may be a consequence of the decreased mitochondrial content and impaired functioning of the ETC. This shift to anaerobic respiration may be a protective response of the placenta to the impaired placental perfusion in PE and so sparing O_2_ for the growing fetus.

### Research Implications

This study is the first to comprehensively characterize and assess mitochondrial content and the regulatory pathways controlling mitochondrial content and function in PE placentas. Moreover, our study may help to explain why despite the strong association of PE with oxidative stress, human studies using systematically acting antioxidants as a treatment for PE are generally unsuccessful [15], and mitochondrial-targeted antioxidants may serve as a better therapeutic target. In addition, our study provides evidence that suggests that abnormalities at the level of the mitochondrion may well be central to key aspects of PE pathophysiology as it is currently well-known that besides their traditional role in energy production, mitochondria are known regulators of apoptosis, oxidative stress, and inflammation, all of which have been heavily implicated in PE pathophysiology. Collectively, this opens up a new avenue of research into the contribution of mitochondrial dysfunction to PE pathophysiology. More research is necessary to unravel which PE-associated factors (e.g., hypoxia) trigger mitochondrial abnormalities in the placenta, in which cell types this is apparent and to what extent this contributes to PE pathophysiology. In addition, whether or not mitochondrially targeted strategies have therapeutic potential in the treatment of PE remains to be discovered in more detail.

### Strengths and Limitations

Our study is the first one that provides a detailed overview of mitochondrial abnormalities and the pathways controlling mitochondrial function and content in PE, which is considered a strength of our study. One obvious limitation, however, is that in our study, we used whole placental tissue homogenates, so it remains to be established in which cell types of the placenta mitochondrial content is affected. Furthermore, a well-recognized difficulty is the lack of non-labored, healthy pre-term control placental samples, as cesarean sections are rarely performed in obstetrically normal pregnancies at gestational age equivalent to those in early onset preeclampsia. Despite the mean gestational age in the PE group of 33 weeks, the group contains also cases of late-onset PE. Due to the relatively small group and the variation of the parameters tested in this study, it was not possible to divide the group into two subgroups (early- and late-onset PE). Based on the findings of Holland et al. [1], the alterations in mitochondria found in our study may even be an underestimation of the presence of mitochondrial abnormalities in early onset PE placentas. Linear regression analysis, however, indicated no significant correlation with all significantly altered parameters found in the PE group compared to the control group in our study and the GA both within the control and PE group (Supplementary Table 3). These findings suggest that GA is not a factor in explaining the alterations in outcome parameters found in the PE group. In line with the similar proportion of labored- and non-labored deliveries in the PE and control group, the mode of delivery did not cause a significant alteration in any of the significantly altered parameters found in the PE group compared to the control group in our study (Supplementary Table 4). In addition, as mitophagy is a flux, changes in protein and mRNA expression levels of these molecules that we assessed in our study are not per se indicative of active or inhibited mitophagy but rather are to be taken as indications of potential changes in the process of mitophagy. The same concept holds true for the process of mitochondrial biogenesis. Moreover, we did not directly assess mitochondrial function by means of respirometry, which prevents hard conclusions regarding (changes in) mitochondrial functionality in PE. Therefore, future studies including measurements of mitochondrial respiration and assessment of actual mitophagy and mitochondrial biogenesis including electron microscopy and recently developed assays for measuring mitophagy [58], e.g., MitoTimer, mt-Keima, and Mito-QC, would provide beneficial knowledge.

## Conclusions

In conclusion, this study demonstrates that PE is associated with significant abnormalities at the level of the mitochondrion in the placenta and that these abnormalities may well be linked to the development of oxidative stress. This implies that mitochondrially targeted antioxidant-based intervention aimed at preventing mitochondrial dysfunction and excessive ROS formation may have therapeutic potential in pregnancy complications like PE.

## Supplementary Information

Supplementary Fig. 1 (PNG 51 kb)

High resolution image (TIF 495 kb)

Supplementary Fig. 2 (PNG 201 kb)

High resolution image (TIF 1338 kb)

Supplementary Fig. 3 (PNG 53 kb)

High resolution image (TIF 585 kb)

ESM 1 (DOCX 50 kb)

ESM 2 (PDF 784 kb)

## Data Availability

All relevant data is implemented in the figures and tables, which can be found within the paper.

## Abbreviations

ABTS: 2,2′-Azino-bis(3-ethylbenzothiazoline-6-sulphonic acid
BAX: Pro-apoptotic Bcl-2-associated X protein
BCL: Anti-apoptotic B cell lymphoma 2
BNIP3: BCL2/adenovirus E1B 19 kDa protein-interacting protein 3
BNIP3L: BCL2/adenovirus E1B 19 kDa protein-interacting protein 3-like
Cat1: Catalase-1
COX: Cytochrome c oxidase
CS: Citrate synthase
DNM1L: Dynamin-related protein 1
ERRα: Estrogen-related receptor alpha
ETC: Electron transport chain
FAO: Fatty acid-β oxidation
Fis-1: Fission 1 protein
FUNDC1: FUN14 domain containing 1
GLUT1: Glucose transporter 1
GSH: Glutathione
GSSG: Glutathione disulfide
HADH: β-Hydroxyacyl-CoA dehydrogenase
HKII: Hexokinase
ISSHP: International Society for the Study of Hypertension in Pregnancy
IUGR: Intrauterine growth restriction
Mfn: Mitofusin
MnSOD2: Manganese-dependent superoxide dismutase
mtDNA: Mitochondrial DNA
NOX: NADPH oxidase
NRF1: Nuclear respiratory factor 1
Opa1: Optic atrophy protein 1
OPTN: Optineurin
PARK2: E3 ubiquitin-protein ligase Parkin
PE: Preeclampsia
PFK: Phosphofructokinase
PGC-1: Peroxisome proliferator-activated receptor gamma coactivator 1
PINK1: PTEN-induced kinase 1
PPAR: Peroxisome proliferator-activated receptor
ROS: Reactive oxygen species
RPL13A: Ribosomal Protein L13a
SOD: Superoxide dismutase
SQSTM1: Sequestosome 1
TEAC: Trolox equivalent antioxidant capacity
Tfam: Transcription factor A
XO: Xanthine oxidase
