# Supplementary figures and images for: Placental Mitochondrial Abnormalities in Preeclampsia
Source: Reprod Sci. 2021 Feb 1;28(8):2186–99. doi: 10.1007/s43032-021-00464-y (PMC8289780; doi:10.1007/s43032-021-00464-y)

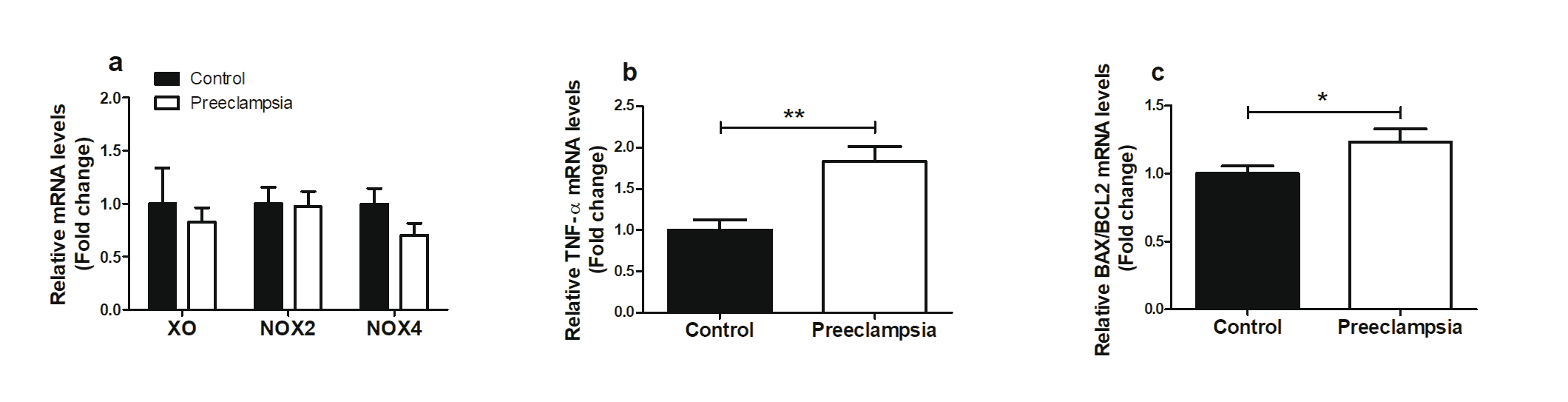

Supplement: Supplementary file 1 — (PNG 51 kb) [file 43032_2021_464_Fig6_ESM.png]

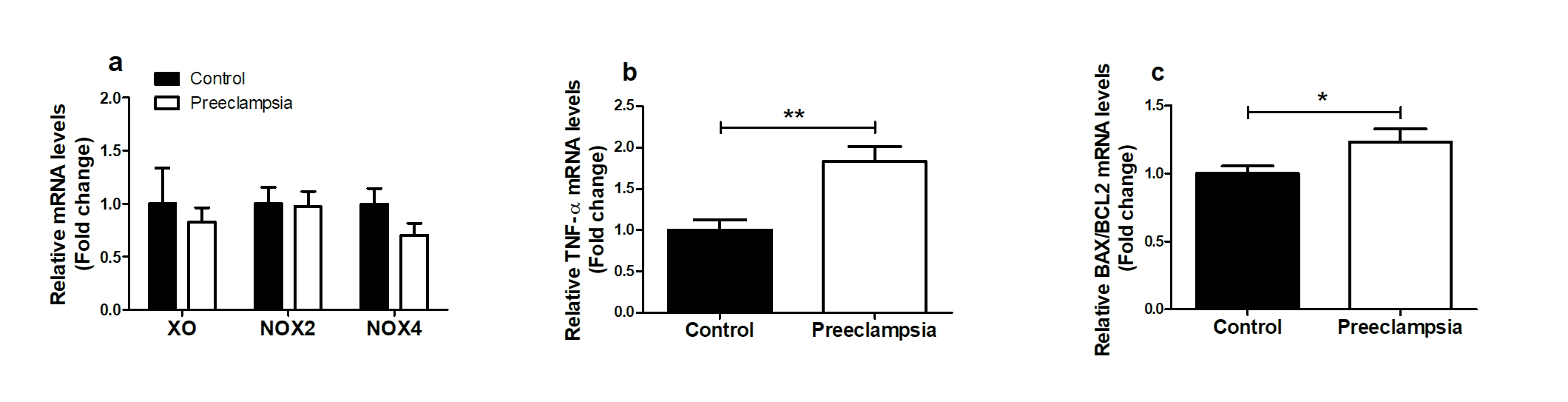

Supplement: Supplementary file 2 — High resolution image (TIF 495 kb) [file 43032_2021_464_MOESM1_ESM.tif]

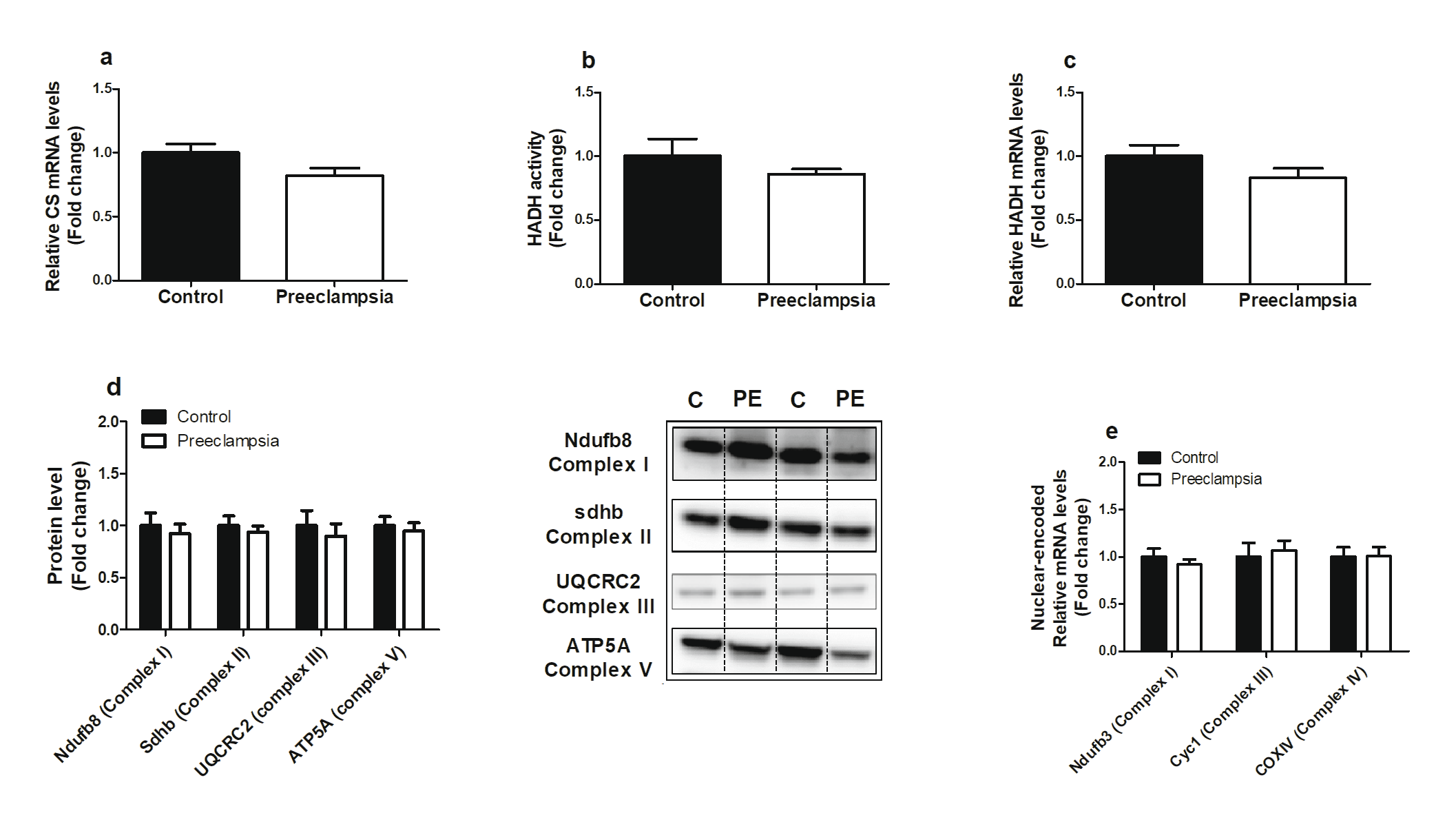

Supplement: Supplementary file 3 — (PNG 201 kb) [file 43032_2021_464_Fig7_ESM.png]

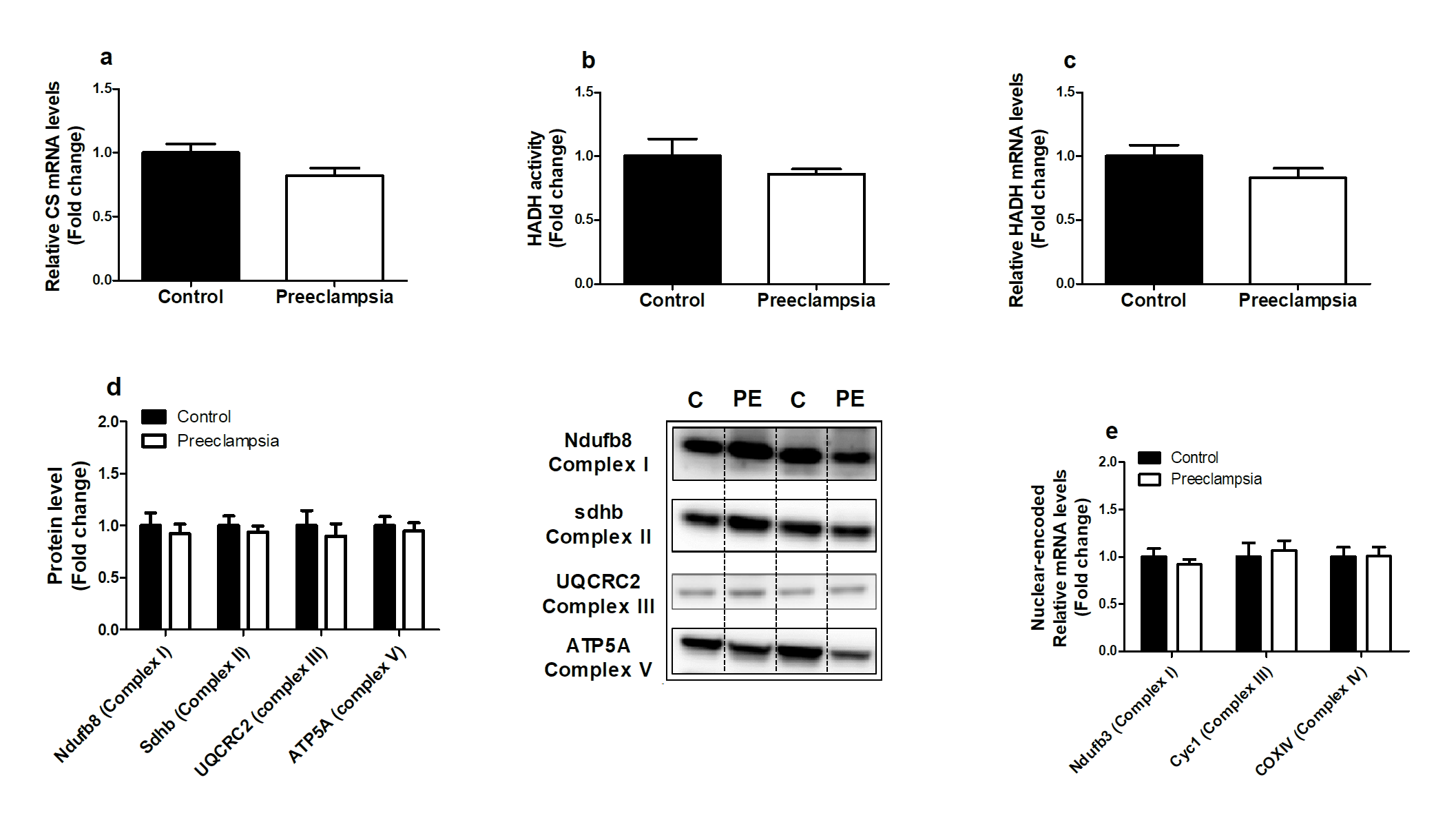

Supplement: Supplementary file 4 — High resolution image (TIF 1338 kb) [file 43032_2021_464_MOESM2_ESM.tif]

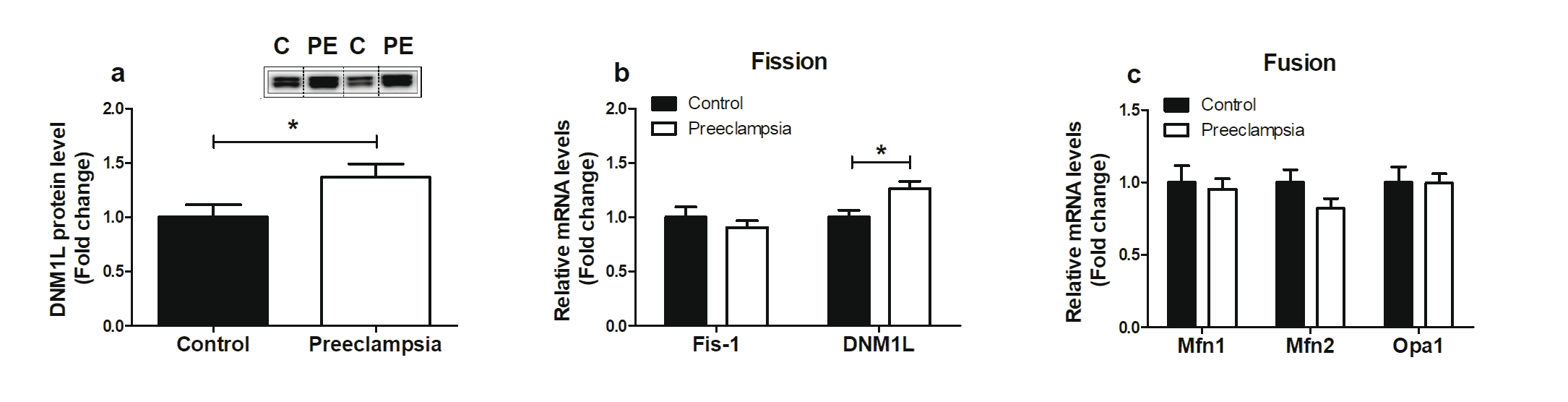

Supplement: Supplementary file 5 — (PNG 53 kb) [file 43032_2021_464_Fig8_ESM.png]

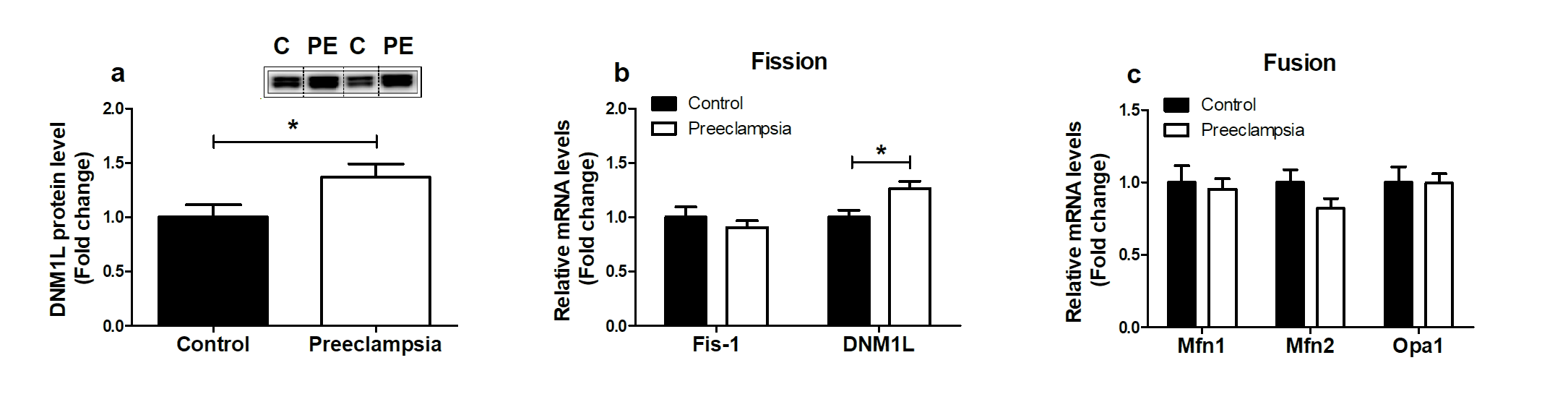

Supplement: Supplementary file 6 — High resolution image (TIF 585 kb) [file 43032_2021_464_MOESM3_ESM.tif]

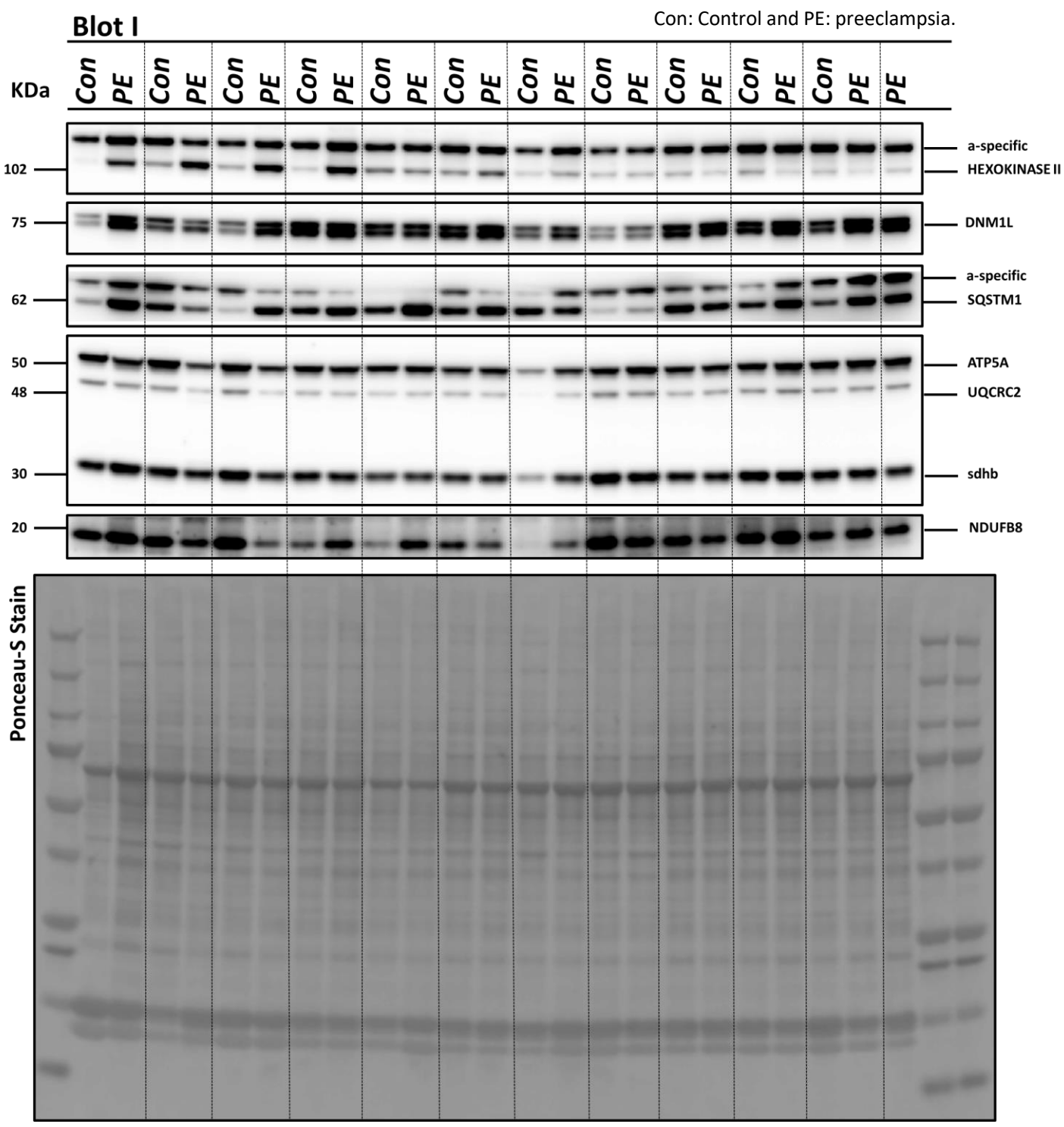

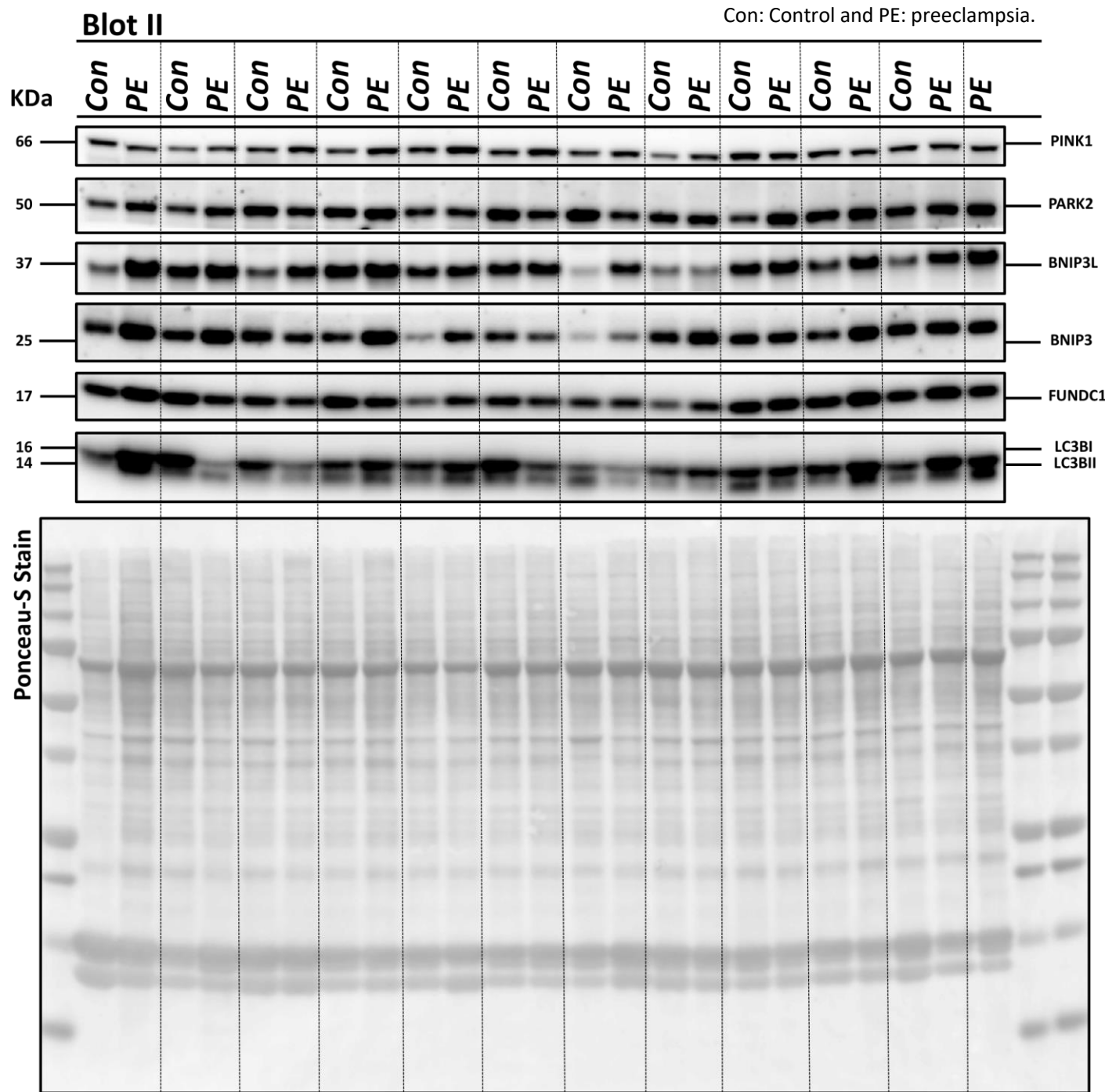

### Blot III

Con: Control and PE: preeclampsia.

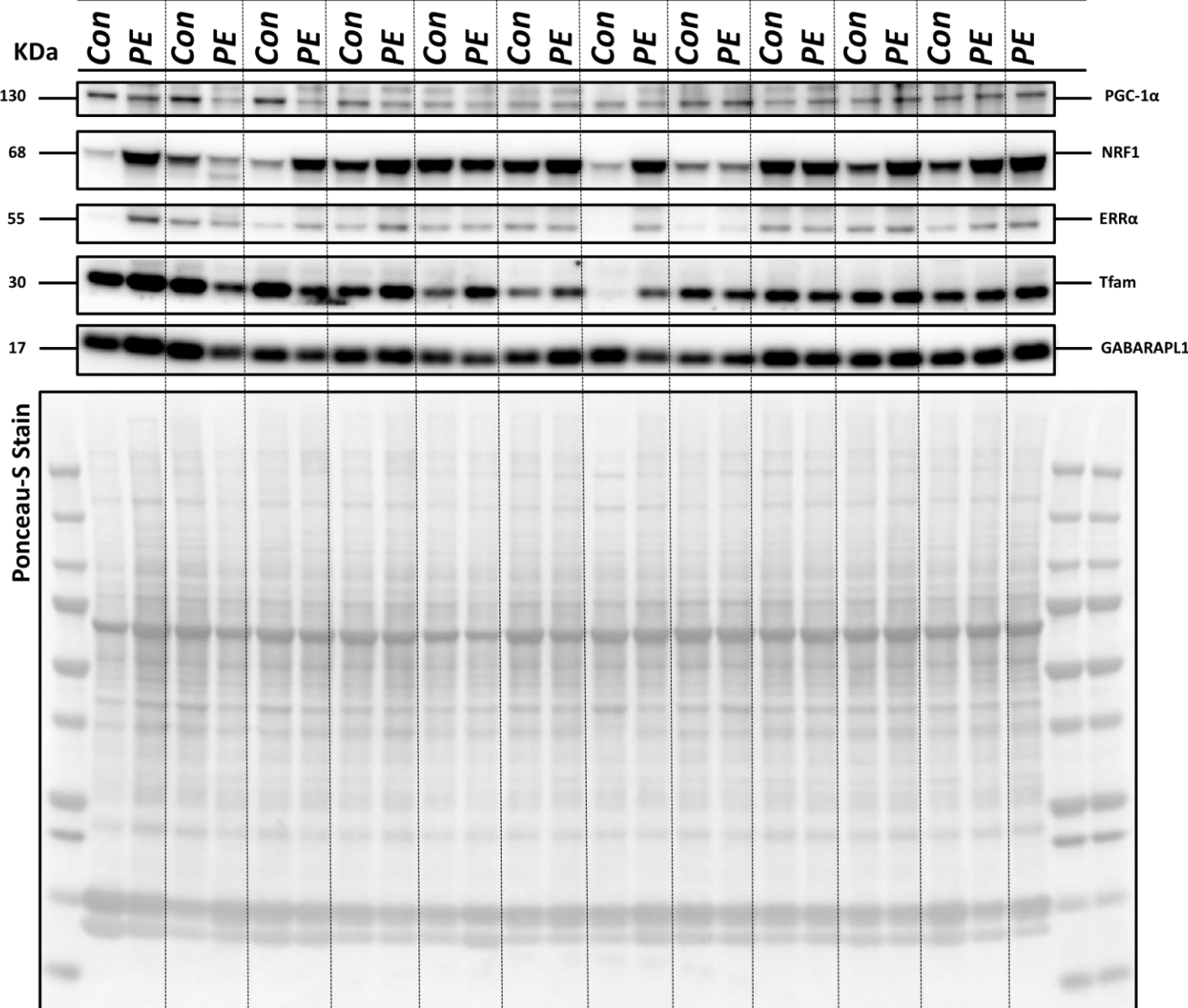

Supplement: Supplementary file 8 — (PDF 784 kb) [file 43032_2021_464_MOESM5_ESM.pdf]
