# Supplementary material for: Placental Mitochondrial Abnormalities in Preeclampsia
Source: Reprod Sci. 2021 Feb 1;28(8):2186–99. doi: 10.1007/s43032-021-00464-y (PMC8289780; doi:10.1007/s43032-021-00464-y)
Supplement: Supplementary file 7 — (DOCX 50 kb) [file 43032_2021_464_MOESM4_ESM.docx]

# Supporting information

## Supporting Figures

### Increased inflammation and apoptosis in PE placentae

mRNA abundance of key constituents of xanthine oxidase and NADPH oxidases were not significantly different in PE placentae compared to controls (Supplementary Fig. 1a). In addition, expression of tumor necrosis factor-α (*TNF-α*) was higher in PE placental tissue (Supplementary Fig. 1b). As mitochondrial processes are closely linked to the coordination of apoptosis, mRNA transcript levels of pro-apoptotic Bcl-2-associated X protein (*BAX*) and the anti-apoptotic B-cell lymphoma 2 (*BCL2*) were measured. An increase in the pro-apoptotic *BAX*/*BCL2* mRNA ratio was observed in PE placentae compared to controls (Supplementary Fig. 1c).

**Supplementary Fig. 1** Increased inflammation and apoptosis in PE placentae. Transcript levels of *XO*, *NOX2* and *NOX4* (**a**), *TNF-α* (**b**) and ratio of *BAX/BCL2* transcript levels (**c**) were assessed in PE as well as control placentae. Data is presented as fold change compared to the control placentae and as mean with SEM from n= 11 (controls), n= 12 (preeclampsia). *p ≤ 0.05, ** p ≤ 0.01. XO: Xanthine oxidase, NOX2: NADPH oxidase 2, NOX4: NADPH oxidase 4, TNF-α: Tumor necrosis factor α, BAX: Pro-apoptotic Bcl-2-associated X protein and BCL: Anti-apoptotic B-cell lymphoma 2.

### Expression of key constituents of mitochondrial metabolic processes in PE placentae.

TCA cycle-related mRNA transcript levels of *CS*, activity and mRNA expression levels of the rate-limiting enzyme of the FAO pathway (HADH) were not significantly different in PE placentae compared to controls (Supplementary Fig. 2a-c). Protein as well as mRNA transcript abundance of key constituents of the electron transport chain (nuclear-encoded OXPHOS sub-units) were not different in PE compared to controls (Supplementary Fig. 2d-e).

**Supplementary Fig. 2** Expression of key constituents of mitochondrial metabolic processes in PE placentae. mRNA transcript levels of *CS* (**a**), HADH enzyme activity (**b**), mRNA transcript levels of *HADH* (**c**) and protein levels of nuclear-encoded OXPHOS sub-units (Ndufb8, Sdhb, UQCRC2 and ATP5A respectively Complex I, II, III and V) (**d**) and mRNA transcript levels of nuclear-encoded OXPHOS sub-units (*Ndufb*, *Cycl-1* and *COXIV* respectively Complex I, III and IV) (**e**) were assessed in PE as well as control placentae. Representative immunoblots are shown and Western blots were corrected for total protein loading assessed by Ponceau S Staining with adjusted contrast equally applied to the whole photograph. Black boxes around the representative pictures indicate that they were cut from the same Western blot. Data is presented as fold change compared to the control placentae and as mean with SEM from n= 11 (controls), n= 12 (preeclampsia) for the mRNA experiments and n= 9 (C: controls), n= 8 (PE: preeclampsia) for the HADH activity assay. Ns: p > 0.05, *p ≤ 0.05. CS: Citrate synthase, HADH: 3-hydroxyacyl-CoA dehydrogenase, Ndufb3: NADH dehydrogenase [ubiquinone] 1 beta subcomplex subunit 3, Sdhb: Succinate dehydrogenase [ubiquinone] iron-sulfur subunit, UQCRC2: Cytochrome b-c1 complex subunit 2, ATP5A: ATP synthase F1 subunit alpha, Cyc1: Cytochrome C1, and COXIV: Cytochrome *c* oxidase subunit IV.

### Increased abundance of mitochondrial fission proteins in PE placentae

Protein levels of dynamin-1-like protein (DNM1L), a key protein involved in mitochondrial fission, were significantly higher in PE placentae (Supplementary Fig. 3a). Furthermore, mitochondrial fission-related mRNA transcript levels of *DNM1L* were also significantly higher in PE while fission 1 (*Fis-1*) transcript levels were unaltered compared to controls (Supplementary Fig. 3b). With regard to mediators of mitochondrial fusion, mRNA transcript levels of mitofusin-1 and 2 (*Mfn1* and *Mfn2*) and mitochondrial Optic atrophy protein 1 (*Opa1*) were not significantly different in PE placentae compared to controls (Supplementary Fig 3c), indicating that specifically mitochondrial fission constituents are higher in PE placentae compared to controls.

**Supplementary Fig. 3** Increased abundance of mitochondrial fission proteins in PE placentae. Fission-related protein levels of DNM1L (**a**), fission-related mRNA transcript levels of *Fis-1* and *DNM1L* (**b**) and fusion-related mRNA transcript levels of *Mfn1*, *Mfn2* and *Opa1* (**c**) were assessed in PE as well as control placentae. Representative immunoblots are shown and Western blots were corrected for total protein loading assessed by Ponceau S Staining with adjusted contrast equally applied to the whole photograph. Black boxes around the representative pictures indicate that they were cut from the same Western blot. Data is presented as fold change compared to the control placentae and as mean with SEM from n= 11 (controls), n= 12 (preeclampsia. *p ≤ 0.05. DNM1L: Dynamin-1-like protein, Fis-1: Fission 1 protein, Mfn1: Mitofusin-1, Mfn2: Mitofusin-2 and Opa1: Optic atrophy protein 1.

## Supporting Tables

**Supplementary Table 1** Primers used for qPCR

| **Target** | **Sense primer (5’-3’)** | **Antisense primer (3’-5’)** |
| --- | --- | --- |
| *Cat1* | GATGTGCATGCAGGACAATCAG | GCTTCTCAGCATTGTACTTGTCC |
| *XO* | AGAAAGTTGGGGCTGAGTGG | GCAGGCATTGGCAGAAAAGT |
| *NOX2* | GACGGCCCAACTGGGATAAT | AGGGTTTCAGCCAAGGCTTC |
| *NOX4* | GTAGGAGACTGGACAGAACGA | ATGAAGGGCAGAATTTCGGAGT |
| *CuZnSOD1* | GGTCCTCACTTTAATCCTCTAT | CATCTTTGTCAGCAGTCACATT |
| *MnSOD2* | TGGACAAACCTCAGCCCTAACG | TGATGGCTTCCAGCAACTCCC |
| *TNF-α* | ATGCCTTTAGATGTGAGCTAACAGTAGGTA | CGTACAGCCATCAAAAAGGGACAC |
| *BAX* | GGTCTTTTTCCGAGTGGCAG | CACAGGGCCTTGAGCAC |
| *BCL-2* | GTCTTTTTCCGAGTGGCAGC | GTAGAAAAGGGCGACAACCC |
| *RPL13A* | CCTGGAGGAGAAGAGGAAAGAGA | TTGAGGACCTCTGTGTATTTGTCAA |
| *COXII* | ACCTGCGACTCCTTGACGTT | GGGGGCTTCAATCGGGAGTA |
| *CS* | GATGTGTCAGATGAGAAGTTACGAGACT | TGGCCATAGCCTGGAACAA |
| *HADH* | TGGCTTCCCGCCTTGTC | TGGAGCCGGTCCACTATCTTC |
| *HKII* | GTAAATACAGTGGATCTCAATCTTCGGG | CAAGGATTTGAGATGATTCGCTATTCA |
| *GLUT1* | TCTGGGCTGCCGGGTTCTAG | TTTGCAGGCTCCCACAGGC |
| *Cycl1* | GCATTCGGAGGGGTTTCCAG | CCGCATGAACATCTCCCCA |
| *COXIV* | CCATGGATGAGAAAGTCGAGT | CGTTCGAGCCCCTGTTCA |
| *PGC-1α* | AAGCCACTACAGACACCGC | TCGTAGCTGTCATACCTGGG |
| *PGC-1β* | CAGAACAAGGAGGCGGAGGTC | AGGTCCAAGTTTGCGAAGC |
| *NRF1* | GCACCTTTGGAGAATGTGGT | CTGGGATAAATGCCCGAAG |
| *NRF2* | CTCACCTGGGAACAGAACAGGAA | ACCCAAGAAATGCAGTCTCGAGC |
| *ERRα* | TGCTGCTCACGCTACCGCTC | TCGAGCATCTCCAAGAACAGC |
| *Tfam* | GAAAGATTCCAAGAAGCTAAGGGTGATT | TCCAGTTTTCCTTTACAGTCTTCAGCTTTT |
| *PPARα* | CAGAACAAGGAGGCGGAGGTC | AGGTCCAAGTTTGCGAAGC |
| *PPARδ* | TGACCAAAAAGAAGGCCCGC | GTCGTGGATCACAAAGGGCG |
| *SQSTM1* | GGTGCACCCCAATGTGATCT | CGCAGACGCTACACAAGTCG |
| *PINK1* | GAAAGCCGCAGCTACCAAGA | AGCACATTTGCGGCTACTCG |
| *PARK2* | GGTTTGCCTTCTGCCGGGAATG | CTTTCATCGACTCTGTAGGCCTG |
| *FUNDC1* | GAAACGAGCGAACAAAGCAG | GCAAAAAGCCTCCCACAAAT |
| *BNIP3* | AGCGCCCGGGATGCA | CCCGTTCCCATTATTGCTGAA |
| *BNIP3L* | CTGCGAGGAAAATGAGCAGTCTCT | GCCCCCCATTTTTCCCATTG |
| *OPTN* | AAGGAGCAACTGGCATTGCA | TCTCCATCAAGGACTGCCTG |
| *GABARAPL1* | ATCGGAAAAAGGAAGGAGAAAAGATC | CAGGCACCCTGGCTTTTGG |
| *LC3A* | CCTGGACAAGACCAAGTTTTTG | GTCTTTCTCCTGCTCGTAGATG |
| *LC3B* | ACCATGCCGTCGGAGAAGAC | TCTCGAATAAGTCGGACATCTTCTACTCT |
| *FIS-1* | CCTGGTGCGGAGCAAGTACAA | TCCTTGCTCCCTTTGGGCAG |
| *DNM1L* | CGACTCATTAAATCATATTTTCTCATTGTCAG | TGCATTACTGCCTTTGGCACACT |
| *Mfn1* | CTGAGGATGATTGTTAGCTCCACG | CAGGCGAGCAAAAGTGGTAGC |
| *Mfn2* | TGGACCACCAAGGCCAAGGA | TCTCGCTGGCATGCTCCAC |
| *Opa1* | TACCAAAGGCATTTTGTAGATTCTGAGTT | GCATGCGCTGTATACGCCAA |
| *Ndufb3* | ACAGACAGTGGAAAATTGAAGGG | GCCCATGTATCTCCAAGCCT |

Cat1: Catalase-1, XO: Xanthine oxidase, NOX2: NADPH oxidase 2, NOX4: NADPH oxidase 4, SOD1: Superoxide dismutase 1, MnSOD2: Manganese-dependent superoxide dismutase, TNF-α: Tumornecrosefactor α, BAX: Pro-apoptotic Bcl-2-associated X protein, BCL: Anti-apoptotic B-cell lymphoma 2, RPL13A: Ribosomal Protein L13a, COXII: Cytochrome c oxidase subunit II, CS: Citrate synthase, HADH: 3-hydroxyacyl-CoA dehydrogenase, HKII: Hexokinase, GLUT1: Glucose transporter 1, Cyc1: Cytochrome C1, COXIV: Cytochrome c oxidase subunit IV, PGC-1α: Peroxisome proliferator-activated receptor gamma coactivator 1-alpha, PGC-1β: Peroxisome proliferator-activated receptor gamma coactivator 1-beta, NRF1: Nuclear respiratory factor 1, ERRα: Estrogen-related receptor alpha, Tfam: Transcription factor A, PPARα: Peroxisome proliferator-activated receptor alpha, PPARδ: Peroxisome proliferator-activated receptor delta, SQSTM1: Sequestosome 1, PINK1: PTEN-induced kinase 1, PARK2: Parkin, FUNDC1: FUN14 domain containing 1, BNIP3: BCL2/adenovirus E1B 19 kDa protein-interacting protein 3, BNIP3L: BCL2/adenovirus E1B 19 kDa protein-interacting protein 3-like and OPTN: Optineurin, GABARAPL1: GABA Type A Receptor Associated Protein Like 1, LC3A: Microtubule-associated protein 1 light chain 3 alpha, LC3B: Microtubule-associated protein 1 light chain 3 beta, Fis-1: Fission 1 protein, DNM1L: Dynamin-related protein 1, Mfn1: Mitofusin-1, Mfn2: Mitofusin-2, Opa1: optic atrophy protein 1 and Ndufb3: NADH oxidoreductase subunit B3.

Supplementary Table 2 Antibodies used for western blot

| **Target** | **RRID** | **Company** | **Product number** | **Dilution** |
| --- | --- | --- | --- | --- |
| HKII | AB_2232946 | Cell Signaling Technology | Cat# 2867 | 1:1000 |
| OXPHOS | AB_2629281 | MitoScience LLC | Cat# MS604 | 1:1000 |
| PGC-1α | AB_10697773 | Millipore | Cat# 516557 | 1:1000 |
| NRF1 | AB_2154534 | Abcam | Cat# ab55744 | 1:1000 |
| ERRα | AB_1523580 | Abcam | Cat# ab76228 | 1:1000 |
| Tfam | AB_10682431 | Millipore | Cat# DR1071 | 1:1000 |
| SQSTM1 | AB_10624872 | Cell Signaling Technology | Cat# 5114 | 1:1000 |
| PINK1 | AB_10127658 | Novus Biologicals | Cat# BC100-494 | 1:2000 |
| PARK2 | AB_2159920 | Cell Signaling Technology | Cat# 4211 | 1:1000 |
| FUNDC1 | AB_10609242 | Santa Cruz Biotechnology | Cat# sc-133597 | 1:500 |
| BNIP3 | AB_2259284 | Cell Signaling Technology | Cat# 3769S | 1:1000 |
| BNIP3L | AB_2688036 | Cell Signaling Technology | Cat# 12396 | 1:1000 |
| GABARAPL1 | AB_2294415 | Proteintech Group | Cat# 11010-1-AP | 1:1000 |
| LC3B | AB_915950 | Cell Signaling Technology | Cat# 2775 | 1:1000 |
| DNM1L | AB_10950498 | Cell Signaling Technology | Cat# 8570 | 1:1000 |

HKII: Hexokinase II, OXPHOS: Oxidative phosphorylation, antibody cocktail (containing NADH: Ubiquinone oxidoreductase subunit B8 (NDUFB8), Succinate dehydrogenase complex, subunit B (SDHB), ubiquinol cytochrome c reductase core protein 2 (UQCRC2), Mitochondrially encoded cytochrome c oxidase I (mt-COI), ATP synthase, H+ transporting, mitochondrial F1 complex, alpha (ATP5A)), PGC-1α: Proliferative activated receptor gamma, coactivator 1 alpha, NRF1: Nuclear respiratory factor 1, ERRα: Estrogen Related Receptor alpha, Tfam: Mitochondrial transcription factor A, SQSTM1: Sequestosome 1, PINK1: PTEN-induced kinase 1, PARK2: Parkin, FUNDC1: FUN14 domain-containing protein 1, BNIP3: BCL2/Adenovirus E1B 19 kDa protein-interacting protein 3, GABARAPL1: γ-aminobutiric acid receptor-associated protein-like 1, LC3B: Microtubule associated protein 1A/1B-light chain 3 beta and DNM1L: Dynamin 1 Like.

Supplementary Table 3 Linear regression analysis on gestational age and all parameters used in this study within the control and PE group.

| **Independent variable** | **Control group** |  |  | **PE group** |  |
| --- | --- | --- | --- | --- | --- |
| **Gestational age** | **Standardized coefficient Beta** | **Sig. (2-tailed)** |  | **Standardized coefficient Beta** | **Sig. (2-tailed)** |
| **Dependent variable** |  |  |  |  |  |
|  |  |  |  |  |  |
| **TEAC** | -0,025 | 0,948 |  | 0,310 | 0,454 |
| **GSSG** | 0,098 | 0,801 |  | -0,495 | 0,121 |
| **mRNA Cat1** | -0,349 | 0,293 |  | -0,470 | 0,171 |
| **mRNA SOD1** | -0,171 | 0,615 |  | 0,131 | 0,685 |
| **mRNA MnSOD2** | 0,323 | 0,333 |  | -0,534 | 0,074 |
| **mtDNA** | -0,268 | 0,486 |  | 0,088 | 0,836 |
| **CS activity** | 0,366 | 0,332 |  | 0,416 | 0,306 |
| **mRNA COXII** | -0,368 | 0,226 |  | -0,061 | 0,859 |
| **PFK Activity** | -0,429 | 0,249 |  | -0,314 | 0,448 |
| **Protein HKII** | -0,105 | 0,759 |  | -0,174 | 0,589 |
| **mRNA HKII** | -0,100 | 0,770 |  | -0,221 | 0,491 |
| **mRNA GLUT1** | 0,164 | 0,629 |  | 0,083 | 0,798 |
| **Protein PGC1α** | -0,094 | 0,784 |  | -0,013 | 0,967 |
| **Protein NRF1** | -0,009 | 0,978 |  | 0,306 | 0,334 |
| **Protein Tfam** | -0,103 | 0,763 |  | 0,213 | 0,507 |
| **Protein ERRα** | 0,086 | 0,802 |  | 0,278 | 0,381 |
| **mRNA PGC1α** | -0,439 | 0,177 |  | 0,030 | 0,930 |
| **mRNA PGC1β** | -0,342 | 0,303 |  | 0,367 | 0,240 |
| **mRNA NRF1** | 0,155 | 0,649 |  | -0,556 | 0,060 |
| **mRNA NRF2α** | -0,362 | 0,274 |  | 0,082 | 0,799 |
| **mRNA Tfam** | -0,226 | 0,504 |  | -0,152 | 0,637 |
| **mRNA ERRα** | 0,155 | 0,649 |  | -0,556 | 0,060 |
| **mRNA PPARα** | -0,419 | 0,199 |  | -0,078 | 0,810 |
| **mRNA PPAR**δ | -0,357 | 0,281 |  | -0,310 | 0,326 |
| **mRNA XO** | -0,186 | 0,584 |  | 0,146 | 0,668 |
| **mRNA NOX2** | -0,024 | 0,947 |  | -0,361 | 0,249 |
| **mRNA NOX4** | -0,021 | 0,951 |  | 0,347 | 0,270 |
| **mRNA TNFα** | -0,350 | 0,321 |  | -0,182 | 0,592 |
| **mRNA BAX/BCL2** | 0,071 | 0,835 |  | -0,272 | 0,392 |
| **mRNA CS** | -0,357 | 0,729 |  | -0,419 | 0,175 |
| **HADH activity** | -0,073 | 0,852 |  | 0,415 | 0,306 |
| **mRNA HADH** | -0,193 | 0,570 |  | 0,102 | 0,753 |
| **Protein Ndufb8** | -0,050 | 0,884 |  | 0,307 | 0,331 |
| **Proetin Sdhb** | -0,043 | 0,900 |  | 0,142 | 0,661 |
| **Protein UQCRC2** | 0,002 | 0,996 |  | 0,087 | 0,789 |
| **Protein ATP5A** | 0,169 | 0,619 |  | 0,255 | 0,423 |
| **mRNA Nsufb3** | 0,570 | 0,085 |  | -0,004 | 0,989 |
| **mRNA Cyc1** | -0,090 | 0,792 |  | -0,191 | 0,553 |
| **mRNA COXIV** | -0,051 | 0,881 |  | -0,165 | 0,608 |
| **Protein DNM1L** | 0,041 | 0,904 |  | 0,336 | 0,286 |
| **mRNA Fis1** | -0,171 | 0,616 |  | -0,350 | 0,264 |
| **mRNA DNM1L** | 0,054 | 0,875 |  | -0,559 | 0,059 |
| **mRNA Mfn1** | 0,023 | 0,946 |  | -0,393 | 0,206 |
| **mRNA Mfn2** | -0,281 | 0,402 |  | -0,372 | 0,234 |
| **mRNA Opa1** | 0,084 | 0,807 |  | -0,432 | 0,161 |
| **Protein PINK1** | -0,132 | 0,698 |  | 0,371 | 0,234 |
| **Protein PARK2** | 0,075 | 0,826 |  | 0,421 | 0,173 |
| **Protein FUNDC1** | 0,207 | 0,542 |  | 0,454 | 0,138 |
| **Protein BNIP3** | 0,450 | 0,164 |  | 0,162 | 0,130 |
| **Protein BNIP3L** | 0,016 | 0,962 |  | 0,522 | 0,082 |
| **mRNA PINK1** | -0,357 | 0,281 |  | -0,572 | 0,066 |
| **mRNA PARK2** | -0,412 | 0,358 |  | -0,135 | 0,799 |
| **mRNA FUNDC1** | -0,369 | 0,264 |  | -0,349 | 0,267 |
| **mRNA BNIP3** | 0,256 | 0,447 |  | -0,107 | 0,741 |
| **mRNA BNIP3L** | -0,265 | 0,432 |  | -0,313 | 0,321 |
| **Protein SQSTM1** | 0,039 | 0,909 |  | 0,512 | 0,089 |
| **Protein GABARAPL1** | -0,056 | 0,870 |  | 0,377 | 0,226 |
| **Protein LC3BI** | 0,263 | 0,435 |  | 0,165 | 0,609 |
| **Protein LC3BII** | 0,220 | 0,516 |  | 0,334 | 0,288 |
| **mRNA SQSTM1** | -0,131 | 0,701 |  | -0,141 | 0,661 |
| **mRNA GABARAPL1** | -0,230 | 0,497 |  | -0,332 | 0,307 |
| **mRNA OPTN** | 0,035 | 0,920 |  | -0,151 | 0,640 |
| **mRNA LC3A** | -0,412 | 0,208 |  | 0,138 | 0,668 |
| **mRNA LC3B** | -0,118 | 0,730 |  | -0,270 | 0,396 |

Cat1: Catalase-1, XO: Xanthine oxidase, NOX2: NADPH oxidase 2, NOX4: NADPH oxidase 4, SOD1: Superoxide dismutase 1, MnSOD2: Manganese-dependent superoxide dismutase, TNF-α: Tumornecrosefactor α, BAX: Pro-apoptotic Bcl-2-associated X protein, BCL: Anti-apoptotic B-cell lymphoma 2, RPL13A: Ribosomal Protein L13a, COXII: Cytochrome c oxidase subunit II, CS: Citrate synthase, HADH: 3-hydroxyacyl-CoA dehydrogenase, HKII: Hexokinase, GLUT1: Glucose transporter 1, Cyc1: Cytochrome C1, COXIV: Cytochrome c oxidase subunit IV, PGC-1α: Peroxisome proliferator-activated receptor gamma coactivator 1-alpha, PGC-1β: Peroxisome proliferator-activated receptor gamma coactivator 1-beta, NRF1: Nuclear respiratory factor 1, ERRα: Estrogen-related receptor alpha, Tfam: Transcription factor A, PPARα: Peroxisome proliferator-activated receptor alpha, PPARδ: Peroxisome proliferator-activated receptor delta, SQSTM1: Sequestosome 1, PINK1: PTEN-induced kinase 1, PARK2: Parkin, FUNDC1: FUN14 domain containing 1, BNIP3: BCL2/adenovirus E1B 19 kDa protein-interacting protein 3, BNIP3L: BCL2/adenovirus E1B 19 kDa protein-interacting protein 3-like and OPTN: Optineurin, GABARAPL1: GABA Type A Receptor Associated Protein Like 1, LC3A: Microtubule-associated protein 1 light chain 3 alpha, LC3B: Microtubule-associated protein 1 light chain 3 beta, Fis-1: Fission 1 protein, DNM1L: Dynamin-related protein 1, Mfn1: Mitofusin-1, Mfn2: Mitofusin-2, Opa1: optic atrophy protein 1 and Ndufb3: NADH oxidoreductase subunit B3.

Supplementary Table 4 The effect of labored or non-labored on and all parameters used in this study.

| **Independent Samples Test** |  |
| --- | --- |
| labored / non-labored |  |
|  | Sig. (2-tailed**)** |
|  |  |
| **TEAC** | 0 .779 |
| **GSSG** | 0 .82 |
| **mRNA Cat1** | 0 .908 |
| **mRNA SOD1** | 0 .161 |
| **mRNA MnSOD2** | 0 .841 |
| **mtDNA** | 0 .465 |
| **CS activity** | 0 .175 |
| **mRNA COXII** | 0 .751 |
| **PFK Activity** | 0 .458 |
| **Protein HKII** | 0 .478 |
| **mRNA HK** | 0 .691 |
| **mRNA GLUT1** | 0 .443 |
| **Protein PGC1α** | 0 .804 |
| **Proetin NRF1** | 0 .925 |
| **Protein Tfam** | 0 .678 |
| **Proetin ERRα** | 0 .252 |
| **mRNA PGC1a** | 0 .354 |
| **mRNA PGC1b** | 0 .837 |
| **mRNA NRF1** | 0 .547 |
| **mRNA NRF2α** | 0 .367 |
| **mRNA Tfam** | 0 .208 |
| **mRNA ERRα** | 0 .547 |
| **mRNA PPARα** | 0 .52 |
| **mRNA PPAR**δ | 0 .718 |
| **mRNA XO** | 0 .355 |
| **mRNA NOX2** | 0 .508 |
| **mRNA NOX4** | 0 .941 |
| **mRNA TNFα** | 0 .275 |
| **mRNA BAX/BCL2** | 0 .549 |
| **mRNA CS** | 0 .944 |
| **HADH activity** | 0 .486 |
| **mRNA HADH** | 0 .234 |
| **Protein Ndufb8** | 0 .608 |
| **Protein Sdhb** | 0 .621 |
| **Protein UQCRC2** | 0 .664 |
| **Protein ATP5A** | 0 .415 |
| **mRNA Nsufb3** | 0 .402 |
| **mRNA Cyc1** | 0 .179 |
| **mRNA COXIV** | 0 .196 |
| **Protein DNM1L** | 0 .767 |
| **mRNA Fis1** | 0 .043 |
| **mRNA DNM1L** | 0 .929 |
| **mRNA Mfn1** | 0 .585 |
| **mRNA Mfn2** | 0 .185 |
| **mRNA Opa1** | 0 .849 |
| **Protein PINK1** | 0 .852 |
| **Protein PARK2** | 0 .934 |
| **Protein FUNDC1** | 0 .097 |
| **Protein BNIP3** | 0 .354 |
| **Protein BNIP3L** | 0 .436 |
| **mRNA PINK1** | 0 .045 |
| **mRNA PARK2** | 0 .073 |
| **mRNA FUNDC1** | 0 .475 |
| **mRNA BNIP3** | 0 .263 |
| **mRNA BNIP3L** | 0 .234 |
| **Protein SQSTM1** | 0 .88 |
| **Protein GABARAPL1** | 0 .429 |
| **Protein LC3BI** | 0 .967 |
| **Protein LC3BII** | 0 .356 |
| **mRNA SQSTM1** | 0 .06 |
| **mRNA GABARAPL1** | 0 .03 |
| **mRNA OPTN** | 0 .376 |
| **mRNA LC3A** | 0 .279 |
| **mRNA LC3B** | 0 .27 |

Cat1: Catalase-1, XO: Xanthine oxidase, NOX2: NADPH oxidase 2, NOX4: NADPH oxidase 4, SOD1: Superoxide dismutase 1, MnSOD2: Manganese-dependent superoxide dismutase, TNF-α: Tumornecrosefactor α, BAX: Pro-apoptotic Bcl-2-associated X protein, BCL: Anti-apoptotic B-cell lymphoma 2, RPL13A: Ribosomal Protein L13a, COXII: Cytochrome c oxidase subunit II, CS: Citrate synthase, HADH: 3-hydroxyacyl-CoA dehydrogenase, HKII: Hexokinase, GLUT1: Glucose transporter 1, Cyc1: Cytochrome C1, COXIV: Cytochrome c oxidase subunit IV, PGC-1α: Peroxisome proliferator-activated receptor gamma coactivator 1-alpha, PGC-1β: Peroxisome proliferator-activated receptor gamma coactivator 1-beta, NRF1: Nuclear respiratory factor 1, ERRα: Estrogen-related receptor alpha, Tfam: Transcription factor A, PPARα: Peroxisome proliferator-activated receptor alpha, PPARδ: Peroxisome proliferator-activated receptor delta, SQSTM1: Sequestosome 1, PINK1: PTEN-induced kinase 1, PARK2: Parkin, FUNDC1: FUN14 domain containing 1, BNIP3: BCL2/adenovirus E1B 19 kDa protein-interacting protein 3, BNIP3L: BCL2/adenovirus E1B 19 kDa protein-interacting protein 3-like and OPTN: Optineurin, GABARAPL1: GABA Type A Receptor Associated Protein Like 1, LC3A: Microtubule-associated protein 1 light chain 3 alpha, LC3B: Microtubule-associated protein 1 light chain 3 beta, Fis-1: Fission 1 protein, DNM1L: Dynamin-related protein 1, Mfn1: Mitofusin-1, Mfn2: Mitofusin-2, Opa1: optic atrophy protein 1 and Ndufb3: NADH oxidoreductase subunit B3.
